# Toward greater realism in inclusive fitness models: the case of caste fate conflict in insect societies

**DOI:** 10.1093/evlett/qrad068

**Published:** 2024-01-11

**Authors:** Helena Mendes Ferreira, Denise Araujo Alves, Lloyd Cool, Cintia Akemi Oi, Ricardo Caliari Oliveira, Tom Wenseleers

**Affiliations:** Department of Biology, Laboratory of Socio-ecology and Social Evolution, Zoological Institute, KU Leuven, Leuven, Belgium; Department of Entomology and Acarology, Luiz de Queiroz College of Agriculture, University of São Paulo, Piracicaba, Brazil; Department of Biology, Laboratory of Socio-ecology and Social Evolution, Zoological Institute, KU Leuven, Leuven, Belgium; Department of Biology, Center of Microbial and Plant Genetics, KU Leuven, Leuven, Belgium; Department of Biology, Laboratory of Socio-ecology and Social Evolution, Zoological Institute, KU Leuven, Leuven, Belgium; Department of Genetics and Evolution, University College London, London, United Kingdom; Departament de Biologia Animal, de Biologia Vegetal i d’Ecologia—Universitat Autònoma de Barcelona, Bellaterra (Barcelona), Spain; Department of Biology, Laboratory of Socio-ecology and Social Evolution, Zoological Institute, KU Leuven, Leuven, Belgium

**Keywords:** caste fate conflict, inclusive fitness theory, social evolution, stingless bees

## Abstract

In the field of social evolution, inclusive fitness theory has been successful in making a wide range of qualitative predictions on expected patterns of cooperation and conflict. Nevertheless, outside of sex ratio theory, inclusive fitness models that make accurate quantitative predictions remain relatively rare. Past models dealing with caste fate conflict in insect societies, for example, successfully predicted that if female larvae can control their own caste fate, an excess should opt to selfishly develop as queens. Available models, however, were unable to accurately predict levels of queen production observed in *Melipona* bees—a genus of stingless bees where caste is self-determined—as empirically observed levels of queen production are approximately two times lower than the theoretically predicted ones. Here, we show that this discrepancy can be resolved by explicitly deriving the colony-level cost of queen overproduction from a dynamic model of colony growth, requiring the incorporation of parameters of colony growth and demography, such as the per-capita rate at which new brood cells are built and provisioned, the percentage of the queen’s eggs that are female, costs linked with worker reproduction and worker mortality. Our revised model predicts queen overproduction to more severely impact colony productivity, resulting in an evolutionarily stable strategy that is approximately half that of the original model, and is shown to accurately predict actual levels of queen overproduction observed in different *Melipona* species. Altogether, this shows how inclusive fitness models can provide accurate quantitative predictions, provided that costs and benefits are modeled in sufficient detail and are measured precisely.

## Teaser

In a fascinating new study, scientists from the Laboratory of Socio-ecology and Social Evolution at the University of Leuven delve into conflicts linked with the choice to become either a queen or a worker in bee societies. Focusing on stingless bees and honeybees, they explore how the ability to control one’s own caste fate can result in many choosing to become queens. In particular, the authors show that in *Melipona* stingless bees, where caste is self-determined, about 10% of all females selfishly opt to become queens, while in related species where caste is nutritionally determined, queens are found to be produced at a rate that aligns with colony needs. The researchers further develop a theoretical model that accurately predicts levels of queen production in these *Melipona* bees, showing that an evolutionary equilibrium is reached when the individual benefit of becoming a queen balances with the collective cost that would occur if too many became queens and too few workers would be produced. This work provides a compelling example of the power of evolutionary theory in making accurate predictions in the field of social evolution, and offers new insights into how conflicts between individual and collective interests play out in animal societies.

## Introduction

Inclusive fitness theory has become one of the cornerstones of modern social evolution theory, and has led to a swathe of successful predictions, in areas ranging from the study of within-organism intragenomic conflict (Gardner & Úbeda, 2017) to understanding patterns of cooperation and conflict in animal societies (Abbot et al., 2011; Frank, 1998; Marshall, 2015). However, outside sex ratio theory (West, 2009), it is still relatively rare for inclusive fitness models to make accurate quantitative predictions. This is not a limitation of inclusive fitness theory per se, but rather caused by the difficulty to accurately model and measure costs and benefits, which is typically much harder than measuring genetic relatedness, and in fact has been achieved in only a handful of cases (Koenig et al., 2023 and references therein). The consequence is that many inclusive fitness models are abstracted to a level where predictions are mostly qualitative as opposed to being formulated at a level where accurate quantitative predictions can be made (Frank, 1998). In some cases, this high level of abstraction may not be due to the uncertainty over the biological specifics, but may be deliberate, for example, if the aim us to capture the essence of a tradeoff or a basic type of behavior or phenomenon that exists across a diversity of taxa that vary in a number of different respects (Abbot et al., 2011), rather than on modeling any one of those taxa with a great deal of realism (Frank, 1998; Parker & Maynard Smith, 1990). Nevertheless, we will argue that this approach also has the downside that it results in models that do not exactly apply to any particular system, and that more specific mechanistic models that rely on accurate empirical data might help improve our understanding of the evolution of specific adaptations and provide more accurate quantitative predictions (Parker & Maynard Smith, 1990).

A good example is provided by models on caste fate conflict in insect societies (Ratnieks, 2001; Wenseleers et al., 2003, 2004a). These models predict that if developing larvae can control their own caste development, an excess should be selected to selfishly develop as queens rather than as workers (Bourke & Ratnieks, 1999; Ratnieks, 2001; Wenseleers et al., 2003, 2004a). By contrast, if the adult workers can control caste development through differential feeding, the expectation is that new queens would be reared more sparingly, aligning with the colony’s requirements. In line with these predictions, only ca. 1 in 10,000 female larvae are reared as queens in honeybees, where queens are approximately twice the size of the workers and caste is nutritionally determined (Wenseleers et al., 2003). In contrast, in stingless bees of the genus *Melipona*, queens and workers are reared in identically sized, mass provisioned cells and caste is self-determined (Bourke & Ratnieks, 1999; Ratnieks, 2001; Wenseleers et al., 2003), resulting in a great excess (around 10%) of all female larvae developing as queens and most being killed or chased out of the colony by the workers soon after emergence (Bueno et al., 2023; Da Silva et al., 1972; Imperatriz-Fonseca & Zucchi, 1995; Santos-Filho et al., 2006; Sommeijer et al., 1994, 2003; Van Oystaeyen et al., 2013; Wenseleers & Ratnieks, 2004; Wenseleers et al., 2004b; 2011). Although qualitatively these data support caste conflict theory, available models have been unable to accurately quantitatively predict levels of queen production in *Melipona* bees (Wenseleers & Ratnieks, 2004). In fact, for most species, the empirically observed levels of queen production (typically between 5% and 15%) (Kerr, 1950; Wenseleers & Ratnieks, 2004) are significantly lower than the theoretically predicted ones (14%−20%, depending on male parentage) (Ratnieks, 2001; Wenseleers et al., 2003).

This discrepancy between theory and observation is likely caused by the unrealistic and highly idealized cost functions assumed in the original models, whereby queen overproduction was posited to cause a directly proportionate, linear reduction in relative colony productivity, measured by swarm and male production (Ratnieks, 2001; Wenseleers et al., 2003). This mirrored an assumption made earlier in Steve Frank’s generic “tragedy of the commons” model (Frank, 1994, 1995), where individual exploitation of common resources was also postulated to cause a proportionate, linear decrease in the success of the group as a whole. Assuming a convex cost function improved the fit of the model (Wenseleers & Ratnieks, 2004; Wenseleers et al., 2003). Nevertheless, this was also unsatisfactory, as ideally one would be able to explain what cost function would be expected a priori, based on a detailed dynamic, microscopic model of how colonies grow and reproduce. The use of abstracted cost/benefit functions that are not mechanistically motivated is also common in other social evolution models (e.g., Dobata, 2012; Lehmann et al., 2008; Olejarz et al., 2015; Reuter & Keller, 2001; Weyna et al., 2021) and is in fact a recognized weakness and common criticism of many published inclusive fitness models (Frank, 1998; Nowak et al., 2010). More broadly, in ecology and evolution in general, it is common for models to be formulated at a purely phenomenological level, and the implied assumptions that are made at a mechanistic level are not always clear (e.g., Brännström & Sumpter, 2005; Stefan et al., 2012).

The aim of this article is twofold. First, we provide a comprehensive meta-analysis of levels of queen production in different *Melipona* species, where caste is self-determined, and compare it with levels of queen production seen in other genera of stingless bees and honeybees, where queens are reared in specialized queen cells and caste is nutritionally determined (Bourke & Ratnieks, 1999; Ratnieks, 2001; Wenseleers et al., 2003). This allows for a clear-cut comparison of the individual and collective optima, enabling us to unambiguously quantify the cost of selfish manipulation of caste fate as well as providing us with solid empirical data to test theoretical predictions. Second, in a theoretical part, we demonstrate how the cost of queen overproduction can be estimated from first principles based on a fully mechanistic population dynamic model that incorporates details of how colonies grow and reproduce and show how this leads to model predictions that match empirical patterns much more accurately. In doing so, we provide a nice example of the power of inclusive fitness theory and its ability to make accurate quantitative predictions on the evolution of conflict and cooperation in biological systems.

## Methods

### Data compilation

Our meta-analysis comprises data on levels of queen production in 43 species of advanced eusocial bees, which includes 21 *Melipona* stingless bees, where caste is self-determined, and 22 species of trigonine stingless bees and the honeybee, *Apis mellifera*, where caste is typically nutritionally determined (see Supplementary Methods for details). To test the model predictions and compute the evolutionarily stable strategy (ESS) levels of queen overproduction in *Melipona*, we collected data on male parentage, the proportion of queen-laid eggs that are female, the life expectancy of the worker bees, and the per capita rates of brood cell construction (for details see Supplementary Tables S1−S5 and Supplementary Methods).

### Model

#### Derivation of the colony-level cost of queen overproduction

To determine the ESS probability with which female larvae should develop as queens if they could control their own caste fate, we first calculate the relative colony-level cost of queen overproduction in terms of reduced swarm and male production from a dynamic model of colony growth and reproduction (for additional background see Supplementary Methods). In line with the biology of *Melipona* stingless bees, the idealized life cycle we consider is one where perennial colonies headed by a single once-mated and outbred queen grow and evenly split into two once they hit a critical number of workers, either due to the colonies swarming or due to them being split in two by beekeepers (Grüter, 2020). In reality, queens may occasionally also be superseded by a daughter queen, but in our model, the exact mode of colony foundation is assumed to not have an impact on the exact colony-level cost of queen overproduction, i.e., both processes are assumed to be roughly equivalent. Considering that queens in *Melipona* stingless bees are produced all year round (Bueno et al., 2023; Moo-Valle et al., 2001), let us denote the probability with which a focal female larva *j* in a colony *i* develops as a queen as *g*_*ij*_ and the colony and population average probabilities with which female larvae develop as queens as *g*_*i*_ and *g*. It is clear that if a female larva would have a higher than average probability of developing as a queen (*g*_*ij*_*/g*_*i*_ > 1) that this would translate into a relative individual benefit, as it would increase its relative chance of inheriting a new swarm. At the same time, if many female larvae would develop as queens (high *g*_*i*_), the productivity of the colony in terms of new swarms and males would go down, as there would be an insufficiently large workforce to sustain the colony. This shows the “tragedy of the commons” nature of caste fate conflict: individually, there is a benefit of developing as a queen, but there would be a cost for all if many did so due to the overexploitation of the colony’s resources and the depletion of its workforce (Wenseleers & Ratnieks, 2004; Wenseleers et al., 2003; 2010). Original caste conflict models assumed a linear, directly proportionate cost, in which the relative colony productivity in terms of male and swarm production would both be given by (1*−g*_*i*_)/(1*−g*) (Ratnieks, 2001; Wenseleers et al., 2003). A biologically more realistic cost function, however, can be derived by explicitly modeling the growth and reproduction of the colony using a dynamic model. If we consider a scenario where a colony headed by a single once-mated queen will split into two once it hits a critical number of workers, and if *b* is the per-capita rate with which workers build and provision new brood cells, which in *Melipona* is roughly constant and largely independent of colony size and unconstrained by the fecundity of the mother queen (Page & Kerr, 1990), *f* is the proportion of those cells that contain female eggs, which is also roughly constant across the year (Bueno et al., 2023; Moo-Valle et al., 2001), *w* is the average probability with which a queen-laid egg is replaced by a male worker-laid egg, *g*_*i*_ is the proportion of the female larvae that develop as queens rather than workers in colony *i*, and *μ* is the daily worker mortality rate, it is clear that the per-capita daily growth in the worker population would be given by $\tau_{W_{i}}=b.f.\left(1-w\right).\left(1-g_{i}\right)-\mu$ (the rate at which new workers are produced minus the rate at which they die) with $W^{\prime }\left(t\right)=\tau_{W_{i}}.W\left(t\right)$, $W\left(t\right)=W\left(0\right).\exp\left(\tau_{W_{i}}.t\right)$ and W(0)=s/2, if we assume s is the size at which colonies swarm and split in two. From this, we can see that the doubling time $T_{d}$ of the worker population of a colony *i* would be equal to $\ln\left(2\right)/\tau_{W_{i}}$ and that the rate at which new swarms could be produced would be given by $S=1/T_{d}$. The relative rate of swarm production by a focal colony *i* relative to an average colony in the entire population can then be written as


$$\begin{array}{l} W_{s}=\frac{S\left(g_{i}\right)}{S\left(g\right)}=\frac{\tau_{W_{i}}}{\tau_{W}}=\frac{b.f.\left(1-w\right).\left(1-g_{i}\right)-\mu }{b.f.\left(1-w\right).\left(1-g\right)-\mu } \end{array}$$ (1)

Given that the relative probability with which a focal female larva *j* in colony *i* would inherit the colony is *g*_*ij*_/*g*_*i*_, the mean relative probability with which that individual would be able to successfully inherit a new swarm would be given by


$$\begin{array}{l} W_{f}=W_{s}\frac{g_{ij}}{g_{i}} \end{array}$$ (2)

In our model, we are not allowing for female larvae to facultatively adjust their probability of becoming a queen in function of the state of the colony, e.g., the amount of food deposited into cells, which might correlate with available food stores and the likelihood for the colony to swarm, even if there is some evidence this might happen (reviewed in Hartfelder et al., 2006). Taking this into account would likely preclude an analytical solution of our model, as it would require the fusion of inclusive fitness theory with optimal control theory (Avila et al., 2021; Day & Taylor, 1997). We also assume that a sufficient number of virgin queens would be available at any one time to allow one to mate and head a daughter swarm and therefore ignore any bet-hedging benefits of producing new queens (Engels & Imperatriz-Fonseca, 1990; Michener, 1974; Sommeijer et al., 1994), as this would be expected to become a significant factor only at much lower rates of queen production than observed in *Melipona*.

Like for swarming, we expect that the reduced worker production caused by queen overproduction would over the long term also negatively impact the rearing of new males. To formally quantify this cost, and considering that males are constantly produced all year round (Moo-Valle et al., 2001), we can observe that the per-capita rate at which new males (*M*) would be produced per day is given by $\tau_{M}=b.\left(w+\left(1-w\right).\left(1-f\right)\right)$, with $M^{\prime }\left(t\right)=\tau_{M}.W\left(t\right)$. If we integrate the total number of males *M*_*T*_ that would be produced over the period between a colony being founded and it swarming (i.e., up to the doubling time *T*_*d*_ for an average colony in the population) we obtain that a focal colony *i* would produce a total number of males in that time of


$$\begin{array}{l} M_{T}=\overset{T_{d}}{\underset{0}{\int }}\tau_{M}.W\left(t\right).dt=W\left(0\right).\left(\exp\left(\tau_{W_{i}}.T_{d}\right)-1\right).\left(\tau_{M}/\tau_{W_{i}}\right) \end{array}$$ (3)

Hence, the relative rate at which new males would be produced over this period by a focal colony *i* relative to an average colony in the population would be


$$\begin{array}{l} W_{m}=\frac{M_{T}\left(g_{i}\right)}{M_{T}\left(g\right)}=\frac{\left(\exp\left(\tau_{W_{i}}.T_{d}\right)-1\right).\tau_{W}}{\left(\exp\left(\tau_{W}.T_{d}\right)-1\right).\tau_{W_{i}}}=\left(2^{\tau_{W_{i}}/\tau_{W}}-1\right).\left(\tau_{W}/\tau_{W_{i}}\right) \end{array}$$ (4)

with $\tau_{W_{i}}=b.f.\left(1-w\right).\left(1-g_{i}\right)-\mu$ and $\tau_{W}=b.f.\left(1-w\right).\left(1-g\right)$−μ.

**Figure 1.**
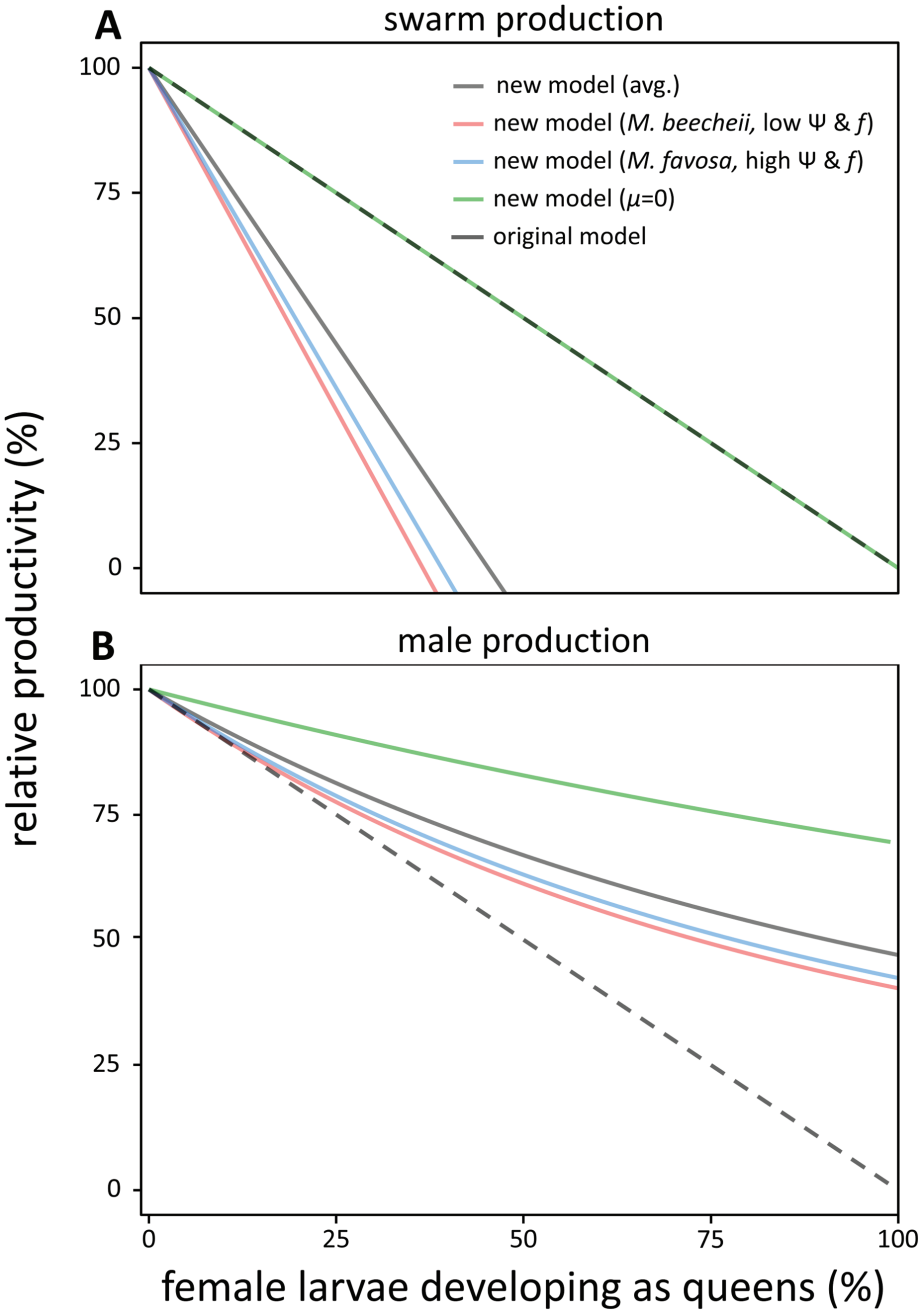
Modelled colony-level cost of queen overproduction in terms of reduced swarm (A) and male production (B). Curves were obtained by plotting expected swarm (*W*_*s*_, Equation 1) and male production (*W*_*m*_, Equation 4) in function of levels of queen production (*g*) divided by the reproductive output if there was no queen overproduction (*g* = 0). For swarm production (A) and for empirically observed average parameter values (Table 2), the cost of queen overproduction is approximately twice as severe as originally assumed (Ratnieks, 2001; Wenseleers et al., 2003), with swarm production already dropping to zero when 45% of all female larvae develop as queens, as opposed to at 100%, assumed in the original models. Only in the absence of worker mortality (*μ* = 0) or with extremely high brood cell building rates (*b* → ∞) would the colony level cost in terms of reduced swarm production reduced to that assumed in the original caste conflict models (Ratnieks, 2001; Wenseleers et al., 2003), but this is evidently highly unrealistic. A similar cost function applies for species with high and low levels of worker reproduction (*M. favosa*: *ψ* = 94.9%, *w* = 16.39%, and *f* = 98.9%; *M. beecheii*: *ψ* = 0.909%, *w* = 0.909%, and *f* = 79.4%), due to the negative covariation between *f* and *w* (Wenseleers et al., 2013). By contrast, for male production, relative output would drop to a lower level when all females would develop as queens, but not to zero as was assumed in the original models (Ratnieks, 2001; Wenseleers et al., 2003), as the original workers that found a colony would still be able to raise males as long as they stayed alive. Hence, in the absence of worker mortality (*μ* = 0) or at very high rates of brood cell construction (*b* → ∞), relative male production would only drop to ln(2) instead of to zero. For further details, see Supplementary Material.

If we plot these inferred cost functions for swarm and male production (Equations 1 and 4) and look at the reproductive output of a focal colony producing a given excess of queens relative to the reproductive output that would be obtained in the optimal situation where queens were not produced in excess (*g* = 0) (Figure 1), we can observe that for both swarm and male production the cost function clearly deviates from the directly proportionate decrease in relative productivity assumed in the earlier models (Ratnieks, 2001; Wenseleers et al., 2003, 2004a, 2010).

Specifically, for swarm production and for empirically estimated parameter values, the cost of queen overproduction is found to be approximately twice as severe as originally assumed (Figure 1A), as swarm production would already be expected to drop to zero when the rate at which new workers are produced would balance with the rate at which they die, i.e., when b.f.(1−w).(1−g) = μ, which for empirically observed parameter values (Table 2) would already occur when 45% of all female larvae would develop as queens, instead of at 100% assumed in the original models (Ratnieks, 2001; Wenseleers et al., 2003, 2004a, 2010). Only in the absence of any worker mortality (*µ* = 0) or at very high rates of cell construction (*b* → ∞) would the cost function approach the linear, proportionate decrease assumed in the earlier models (Figure 1A, green and black dashed lines), but this would clearly be very unrealistic (Ratnieks, 2001; Wenseleers et al., 2003, 2004a, 2010). If we plot the cost function using species-specific parameter estimates (Table 2), we see that a similar colony-level cost functions apply in species with high (*Melipona favosa*) and low levels of worker reproduction (*Melipona beecheii*) (high and low *ψ* and *w*, Figure 1A, blue and red lines). This is due to the fact that queens lay relatively more female eggs (higher *f*) in species with more worker reproduction, causing f.(1−w) and the net cost of queen overproduction in terms of reduced swarm production to stay approximately constant. Earlier, queen-worker coevolution among these traits (*f* and *w*) has been studied in detail in Wenseleers et al. (2013).

**Table 2.** Summary of individual model parameters (*ψ, f*, *w*, and *µ*) estimates for eight *Melipona* species together with 95% confidence intervals (cf. Supplementary Tables S1–S4) together with observed and predicted ESS percentages developing as queens (*g* and g*) (cf. Supplementary Table S5 and Equation 6). The per-species value of *w* was calculated based on the observed percentages of workers’ sons *ψ* and the percentage of queen-laid eggs that were female *f* and was given by (1 − *f*).*ψ*/ (1 − *f*.*ψ*) (with 95% confidence intervals calculated via parametric bootstrapping). As there was missing data on the reproductive efficiency *b* for many of the eight study species, we used the average reproductive efficiency *b* = 0.049 [0.041−0.058] observed across all species throughout (Supplementary Table S4). Population prediction intervals on the predicted ESS per species and overall average predicted ESS were calculated via parametric bootstrapping, by resampling the individual parameters from a multivariate normal distribution that was parameterized based on the coefficients and variance-covariance matrix of the fit used to estimate each parameter (for details see Supplementary R script). The ESS predicted by the old original models of Ratnieks (2001) and Wenseleers et al. (2003) are also shown for comparison.

Species	Percentage workers’ sons (*ψ*)	Percentage queen-laid eggs that are female (*f*)	Percentage queen-laid eggs replaced by male worker-laid egg (*w*)	Mean worker mortality rate per day (*µ*)	Observed percentage females developing as queens (*g*)	Predicted ESS percentage of females developing as queens (g*)	Old model predictions (g*_old_)
*M. asilvai*	19.9% [5.68%−50.6%]	88.1% [87.1%−89.1%]	2.84% [0.73%−10.58%]	0.0244 [0.0238%−0.0252]^a^	7.22% [4.10%−12.42%]	9.21% [3.77%−13.40%]	19.15% [17.54%−19.78%]
*M. beecheii*	0.909% [0.0552%−13.2%]	79.4% [78.8%−80.0%]	0.19% [0.01%−3.44%]	0.0194 [0.0182%−0.0207]	11.23% [7.80%−15.91%]	11.29% [7.39%−13.53%]	19.96% [19.39%−20.00%]
*M. bicolor*	37.5% [28.0%−48.1%]	96.3% [96.0%−96.5%]	2.20% [1.35%−3.60%]	0.0227 [0.0207%−0.0249]	8.68% [5.30%−13.91%]	11.03% [7.39%−13.53%]	18.31% [17.70%−18.79%]
*M. favosa*	94.9% [92.9%−96.4%]	98.9% [98.8%−99.1%]	16.39% [11.54%−22.73%]	0.0246 [0.0231%−0.0264]	7.80% [4.32%−13.69%]	7.59% [4.47%−9.96%]	14.69% [14.58%−14.85%]
*M. marginata*	37.7% [19.9%−59.7%]	92.5% [91.8%−93.2%]	4.37% [1.72%−10.33%]	0.0243 [0.0222%−0.0267]	10.06% [6.13%−16.06%]	9.52% [5.05%−9.96%]	18.30% [17.04%−19.18%]
*M. quadrifasciata*	63.6% [43.0%−80.2%]	97.4% [97.1%−97.7%]	4.31% [2.01%−8.98%]	0.0244 [0.0238%−0.0252]^a^	7.04% [4.33%−11.26%]	9.69%[4.32%−13.19%]	16.85% [15.78%−18.02%]
*M. scutellaris*	23.3% [19.3%−27.7%]	88.2% [87.9%−88.4%]	3.47% [2.74%−4.37%]	0.0228 [0.0208%−0.0250]	8.24% [6.03%−11.17%]	9.99% [6.14%−12.55%]	19.00% [18.79%−19.18%]
*M. subnitida*	35.6% [31.2%−40.1%]	97.2% [96.7%−97.7%]	1.50% [1.17%−1.94%]	0.0244 [0.0238%−0.0252]^a^	7.61% [4.59%−12.34%]	10.41 [5.49%−13.93%]	18.41% [18.17%−18.62%]
***Avg.***	**33.75%[24.90%−43.92%]**	**94.57% [94.37%−94.77%]**	**2.62%[0.055%−18.94%]**	**0.0245[0.0238–0.0252]**	**8.39%[7.08%−9.93%]**	**9.97%[4.91%−13.48%]**	**18.50% [14.64%−19.99%]**

^a^Species-specific estimate not available; overall average across different *Melipona* species used instead.

For the cost of queen overproduction on male production, by contrast, we find that for empirically observed average parameter values, relative male production would not drop all the way to zero if all females developed as queens, but that it would still be 47% of the optimal maximal productivity, which is due to the contribution of the workers that would found a colony, and similar values are obtained if we use species-specific parameter values for species with high (*M. favosa*) or low levels of worker reproduction (*M. beecheii*) (Figure 1B). This implies that in earlier models this cost was overestimated (Ratnieks, 2001; Wenseleers et al., 2003). In the unrealistic case with zero worker mortality (*µ* = 0) and with all females developing as queens, productivity would remain at ln(2) = 69% of optimal maximal productivity.

#### ESS level of queen overproduction

With our newly derived functions for $W_{f}$ and $W_{m}$ (Equations 2 and 4), it is straightforward to calculate the selection differential *σ* on the probability with which females should develop as queens g. In particular, from kin selection theory, using a neighbor-modulated fitness framework (Frank, 1998), and assuming that many female larvae within a given colony would compete to become queens, the selection differential is given by (cf. Wenseleers et al., 2003, 2010):


$$\begin{array}{lll} \sigma = & \;c_{f}.\frac{\partial W_{f}}{\partial g_{ij}}.1+c_{f}.\frac{\partial W_{f}}{\partial g_{i}}.r_{f}+c_{m}.\frac{\partial W_{m}}{\partial g_{i}}.r_{m}\propto \frac{\partial W_{f}}{\partial g_{ij}}\; & +\frac{\partial W_{f}}{\partial g_{i}}.r_{f}+\frac{c_{m}}{c_{f}}.\frac{\partial W_{m}}{\partial g_{i}}.r_{m} \end{array}$$ (5)

where the relatedness of developing female larvae to the males reared in the colony $r_{m}$ would in presence of worker reproduction be a weighted average of the relatedness to queen-produced brothers and worker-produced nephews, $r_{m}=r_{brothers}.\left(1-\psi \right)+r_{nephews}.\psi$, if the proportion of males that are workers’ sons ψ=w/((1−w).(1−f)+w) and where due to haploidy and the fact that colonies are typically headed by a single-mated queen, and assuming random matting, $r_{f}=r_{sisters}=3/4$, $\;r_{brothers}=1/2$, $r_{nephews}=r_{sisters}$, and given class-specific reproductive values of males and females (queens) $c_{m}$ and $c_{f}$, $c_{m}/c_{f}=1/\left(2-\psi \right)$ (Table 1) (Crozier & Pamilo, 1996). Calculating the partial derivatives in Equation 5, corresponding to the costs and benefits of developing as a queen with a slightly increased probability in the limit of weak selection $\left(g_{ij}\to g_{i}\to g\right)$, and solving for the value of g where the selection differential would equal zero then yields the ESS probability for female larvae to develop as queens if they can determine their own caste fate:

**Table 1.** Parameters and terminology used in the model.

Parameters and terminology	
*t*	Time in days
*W(t)*	Total number of workers present at time *t*
*M(t)*	Total number of males present at time *t*
*g*_*ij*_	Probability that an individual focal larva *j* in a focal colony *i* develops as a queen
*g*_*i*_	Average proportion of the individual female larvae that develop as queens in colony *i*
*g*	Proportion of the female larvae that develop as queens on average in the whole population
$g^{*}$	ESS proportion of female larvae that would be expected to develop as queens
*b*	Number of new cells provisioned per worker per day
*f*	Proportion of the queen’s eggs that are female
*w*	Average probability with which a queen-laid egg is replaced by a male worker-laid egg
*L*	Worker life expectancy in days (= 1/*μ*)
*μ*	Daily mortality rate of workers (= 1/*L*)
*s*	Colony size (number of workers) at which a colony would swarm and split in two
ψ	Proportion of the adult males that are workers’ sons (= *w*/((1 − *w*).(1 − *f*) + *w*))
*c*_*m*_	Class-specific reproductive value of males, i.e., product of the stable frequency of males and their individual reproductive value *v*_*m*_
*c*_*f*_	Class-specific reproductive value of females (queens), i.e., product of the stable frequency of females (queens) and their individual reproductive value *v*_*f*_
*r*_*m*_	Regression relatedness to males reared in the colony
*r*_*f*_	Regression relatedness to sister queens reared in the colony


$$\begin{array}{l} g^{*}=\frac{\left(1-r_{f}\right).\left(b.f.\left(1-w\right)-\mu \right)}{b.f.\left(1-w\right).(1-\left(c_{m}/c_{f}\right).r_{m}.\left(1-ln\left(4\right)\right)} \end{array}$$ (6)

## Results

### Quantitative fit to empirical data

**Figure 2.**
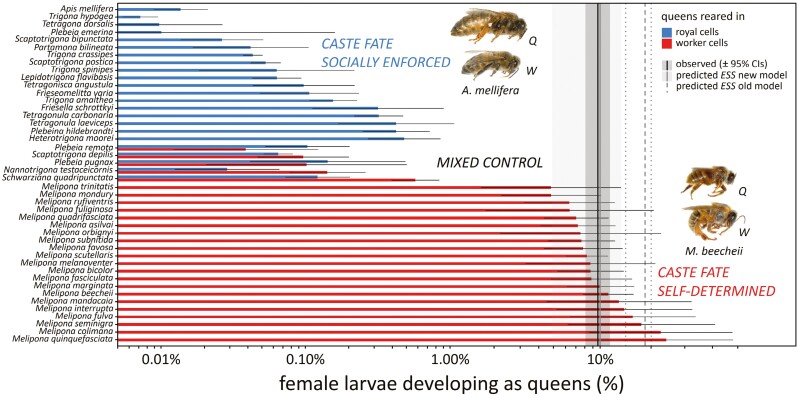
Empirical test of ESS model predictions based on levels of queen production in stingless bees and honeybees with or without individual control over caste development. In *Melipona* stingless bee species queens are reared in worker cells, and caste is under individual control, which contrasts with species where queens are reared in larger royal cells and caste fate is socially enforced via differential feeding. In the group of stingless bee species shown in the middle, queens can be reared in both worker cells and royal cells, implying caste fate is under mixed control. In support of caste conflict theory and our new ESS model, self-determination of caste fate results in two orders of magnitude higher levels of queen production and queens therefore being produced in great excess of colony needs. The black vertical line and dark gray shaded area represent the observed overall average percentage of female larvae developing as queens in the 21 *Melipona* species and 95% confidence intervals (9.65% [8.07%−11.50%]). The gray vertical line and light gray shaded area represent the percentage of female larvae that would be expected to develop as queens, based on parameter estimates from eight *Melipona* species (Table 2) and our current ESS model and 95% population prediction intervals (9.97% [4.91%−13.48%]). Dashed black lines represent the percentage of female larvae that were expected to develop as queens according to the previous model of Ratnieks (2001) and Wenseleers et al. (2003), which was derived under the assumption of a linear, directly proportionate cost of queen overproduction. Earlier model predictions overestimated the % of females that should develop as queens by a factor of 2, while our current model, which accurately derives the expected colony-level cost function, closely matches empirical data. For details see Tables 2 and Supplementary Table S5. Picture credits: Tom Wenseleers.

If we plug empirical estimates of our model parameters obtained from eight species of *Melipona*, where queens and workers are reared from the same types of brood cells and caste is self-determined, into our ESS equation (Equation 6), we find that the average predicted ESS is for 9.97% ([4.91%−13.48%] 95% PIs) of the female larvae to develop as queens. This prediction closely aligns with the overall average observed percentage of 8.39% ([7.08%–9.93%] 95% CIs) developing as queens in these eight species (Table 2), and with the 9.65% ([8.07%−11.50%] 95% CIs) developing as queens in a larger set of 21 *Melipona* species (Supplementary Table S5, Figure 2). These model results therefore fit the empirical data much better than the original caste conflict model, which predicted that between 14% and 20% of all female larvae should develop as queens, depending on male parentage (18.50% [17.98%−18.88%] 95% PIs given the observed values of ψ, cf. Table 2 and Figure 2) (Ratnieks, 2001; Wenseleers et al., 2003). The fact that the predicted ESS is approximately twofold lower than the original one is also in line with the fact that the inferred cost of queen overproduction on swarm production turned out to be approximately twice as severe as originally assumed (Figure 1A).

If we compare levels of queen production in *Melipona* with those observed in other trigonine stingless bee genera and in honeybees, where queens are much larger than the workers and caste fate is socially enforced via differential feeding, we can unambiguously confirm that queens in *Melipona* are produced greatly in excess of colony needs, as the average percentage of females developing as queens is more than two orders of magnitude higher in *Melipona* (9.65% [8.07%−11.50%] 95% CIs) than in other trigonine stingless bees and honeybees (0.07% [0.05%−0.09%] 95% CIs, Figure 2 and Supplementary Table S5). Hence, we can safely reject other previously proposed alternative theories for excess queen production in *Melipona*, such as it being a bet hedging strategy to guard against accidental queen loss (Bourke & Ratnieks, 1999; Engels & Imperatriz-Fonseca, 1990; Michener, 1974; Sommeijer et al., 1994; Wenseleers et al., 2004b), as that should apply equally to trigonine stingless bees and clearly does not explain the scale of the observed excess.

In between the two outcomes where caste is either self-determined (*Melipona*) or socially enforced (most other trigonine stingless bee genera), there is also a set of stingless bee species where queens can be reared both in royal cells and in worker cells (reviewed in Hartfelder et al., 2006; Ribeiro et al., 2006) (Figure 2). In these cases, developing as a miniature queen from a worker cell has been interpreted as a selfish “cheater” strategy aimed at evading an intended worker fate (Ribeiro et al., 2006; Wenseleers et al., 2004a, 2005), as both types of queens—regular queens and miniature queens—can successfully head daughter colonies (Ribeiro et al., 2006). In addition, in some of these species, miniature queens are overproduced relative to regular queens, e.g., in *Schwarziana quadripuctata* (percentage of females reared as miniature queens in worker cells: 0.58% [0.40%−0.84%] vs. those reared as regular queens from royal cells: 0.12% [0.07%−0.20%]) (Figure 2 and Supplementary Table S5, Santos-Filho et al., 2006).

### Sensitivity analysis

**Figure 3.**
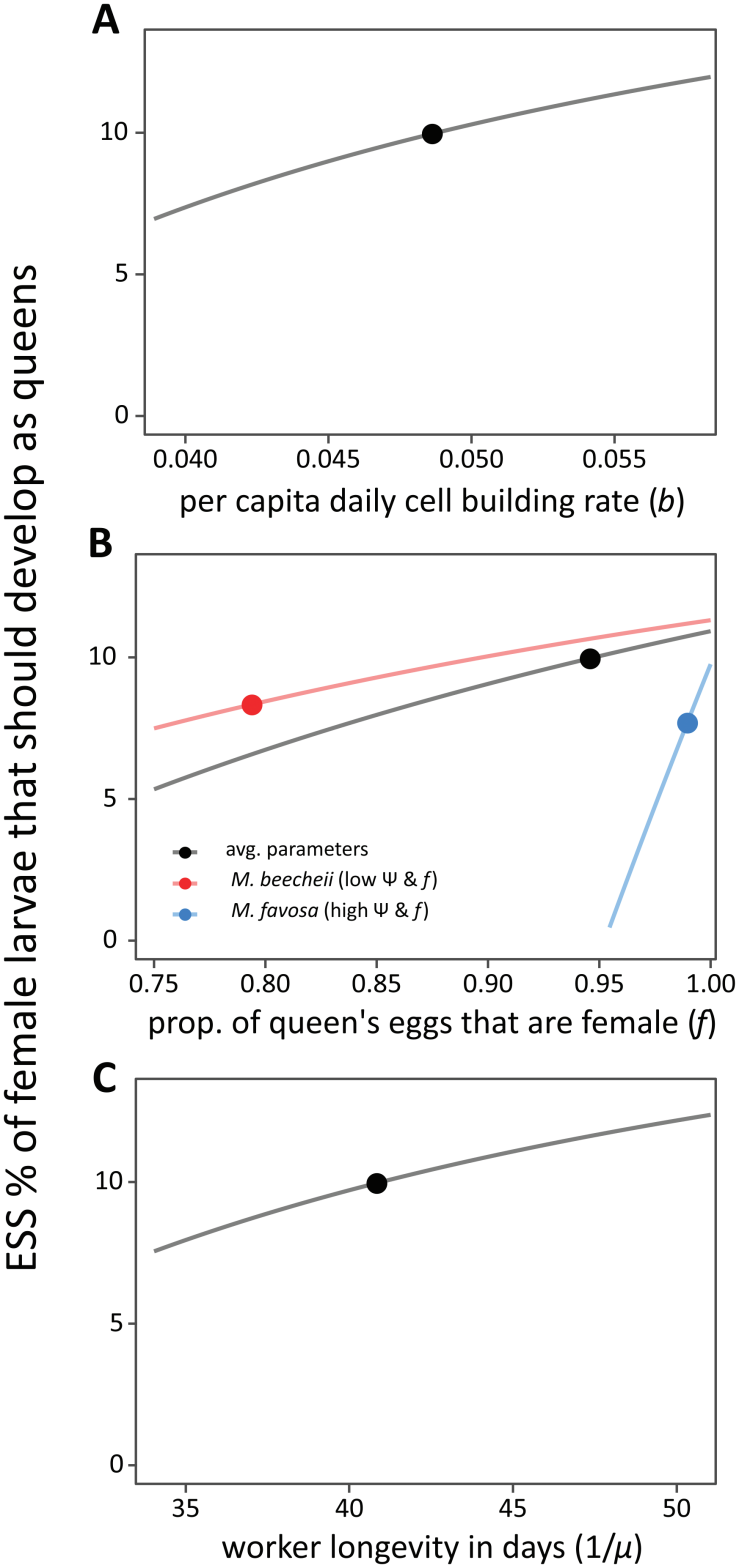
Predicted effect of model parameters on ESS level of queen production. Curves show the predicted partial effect of varying each individual parameter, holding constant the remaining parameters; points are estimates at average parameter values or at parameter values observed in species with high or low levels of worker reproduction (cf. Table 2). (A) A higher per capita daily cell building rate (*b*) results in a reduced colony-level cost of queen production and hence a higher ESS queen production, (B) queens laying a higher proportion of female eggs (*f*) results in the development of more workers and leads to a lower colony-level cost of queen overproduction and hence a higher ESS queen production, but with a more pronounced effect in species with high levels of worker reproduction, and (C) increased worker longevity (*L* = 1/*µ*) leads to a reduced colony-level cost of queen overproduction and hence a higher ESS queen production.

Looking at the species-specific ESS estimates, we can see that the predicted range for females to develop as queens in *Melipona* (between 7.6% in *M. favosa* and 11.3% in *M. beecheii*) closely matches the predicted range among those eight species (7.0%−11.2%). Owing to limited sample sizes, uncertainty in the measured parameters and unavailability of some of the parameters for individual species (which sometimes forced us to then use mean values observed for other species), both the species-specific estimates as well as the theoretically predicted ESS values per species show relatively large confidence intervals (Table 2). This precludes meaningful analysis at the individual species level, e.g., to determine how variation in each parameter affects the observed level of queen overproduction. At a theoretical level, however, it is straightforward to perform a sensitivity analysis of our ESS result (Figure 3).

First, from a relatedness angle, based on Equation 6, a lower relatedness among developing female larvae $\left(r_{f}\right)$ would be expected to favor more larvae to develop as queens. Empirically, however, stingless bees hardly show any variation in sister-sister relatedness, as with the exception of *Melipona bicolor* (Bego, 1983, 1989; Imperatriz-Fonseca & Zucchi, 1995)—where multiple breeding queens sometimes co-occur, though with effective maternity still being close to 1 (Alves et al., 2011)—all species are headed by a single once-mated queen, resulting in $r_{f}≅3/4$. Worker reproduction also affects relatedness patterns, though it has a dual effect, as the replacement of queen-laid eggs by worker-laid eggs both increases the cost of queen overproduction and increases the relatedness to the males that are reared $\left(r_{m}\right)$ (Wenseleers & Ratnieks, 2004; Wenseleers et al., 2003), hence causing the cost of queen overproduction to fall on closer relatives (Wenseleers & Ratnieks, 2004). Both effects would imply that naively, one would expected higher level of worker reproduction to result in lower levels of excess queen production (Figure 3). In practice, the situation is more complex, however, due to the fact that the proportion of female eggs laid by the queen (*f*) negatively covaries with levels of worker reproduction (Table 2), likely as part of a coevolutionary process whereby the queen tries to limit the cost of worker reproduction or workers reproduce more when the mother queen lays more female eggs (Wenseleers et al., 2013). This partly compensates the expected increased cost of queen overproduction in species with higher levels of worker reproduction. Most straightforward to analyse are the effects of the other demographic parameters on the expected ESS, which all influence the expected colony-level cost of queen overproduction in a simple and intuitive way (Figure 3). In particular, a higher per capita rate of brood cells construction (*b*), queens laying a higher proportion of female eggs (*f*), and workers having a lower mortality (*µ*) would all reduce the colony-level cost of queen overproduction and hence result in a higher ESS level of queen production (Figure 3A–C). Detailed tests of these predictions, however, would require accurate estimates of these parameters for a larger set of species, which are not straightforward to obtain.

## Discussion

Overall, our analysis on caste conflict in insect societies provides an interesting case study to show how inclusive fitness theory can be applied to make precise predictions on the balance between cooperation and conflict in a biological system. While previous models on this were instructive in demonstrating the basic structure of caste fate conflict (Ratnieks, 2001; Wenseleers et al., 2003), they were in many ways mere toy models, due to the unrealistic linear, directly proportionate cost function that they assumed, which they shared with Frank’s equally abstract tragedy of the commons model (Frank, 1995). In the present study, we modeled this cost function explicitly, based on a fully dynamic model of colony growth and reproduction, and used it to derive an ESS result giving the exact expected percentage of females that would be selected to become queens if they had the power to do so. Empirical data from *Melipona* bees, where caste is self-determined (Bourke & Ratnieks, 1999; Ratnieks, 2001; Wenseleers et al., 2003), were shown to accurately match these predictions, with observed levels of queen production closely aligning with the theoretically predicted values, thereby significantly improving previous models, which overestimated the actual values by a factor of almost 2 (Figure 2). Presumably, the rearing of queens from worker cells in *Melipona* originated as a cheater strategy (Ribeiro et al., 2006; Wenseleers et al., 2004a), with phylogenetic evidence suggesting that *Melipona* secondarily lost the ability to rear regular queens in royal cells, perhaps because of the numerical advantage of miniature queens, which made it no longer worth it for the workers to invest in the construction of royal cells to rear regular queens (Rasmussen & Cameron, 2010). Selective elimination of miniature queens by the workers or preferential storage of regular queens in imprisonment chambers, however, may act as a policing mechanism that would reduce the incentive for female larvae to develop as miniature queens, thereby preventing the spread of this selfish trait and keeping ESS levels of miniature queen production at a low level (Wenseleers et al., 2004a).

As a whole, accurate quantitative predictions as derived here are rare in the field of social evolution, and have been mainly restricted to sex ratio theory (Gardner et al., 2012; West, 2009). In fact, even in that field it has taken decades to go from simple models that predicted optimal sex allocation patterns in insect societies based only on relatedness asymmetries (Trivers & Hare, 1976) to ones that specifically took into account the actual mechanisms and costs of sex ratio manipulation (West, 2009). Our model should therefore address the common critique that inclusive fitness models frequently lack enough mechanistic detail (Nowak et al., 2010). We would also argue that this criticism does not apply specifically to inclusive fitness models, but rather relates to the tradeoff that is made between the level of abstraction and realism in any model (Shou et al., 2015). In fact, Ratnieks (2001) had also assumed a simple linear one-on-one cost function, despite having used a formal population genetic approach. The motivation in that case to refrain from making a more complex model was simply that at the time, it seemed doubtful that the required demographic parameters that would go into a more detailed model would be empirically available. As we showed, this was not entirely warranted, since at least a simple model of colony growth could be parameterized based on readily available literature data. In fact, over the last decade, there has been a general trend for models in the social evolution literature to always become ever more complex and biologically realistic, e.g., taking into account the coevolution among several traits (e.g., Quiñones & Pen, 2017; Quiñones et al., 2020; Wenseleers et al., 2013), incorporating detailed life cycles and preadaptations (Quiñones & Pen, 2017; Wenseleers et al., 2013), generalizing Hamilton’s rule to stochastic or otherwise dynamic environments (e.g., Kennedy et al., 2018) or using individual-based simulations to take into account details of the underlying genetic architecture of the trait under study as well as critical stochastic aspects of the problem under study (e.g., Davies & Gardner, 2018; Kennedy et al., 2021; Kreider et al., 2021).

Usually, the level of detail at which a problem is modeled is tailored to the level at which parameters can be empirically measured, however. In that sense, it is worth mentioning that the approach we used here that allowed us to indirectly quantify the costs and benefits of queen overproduction based on a dynamic model of colony growth is much more feasible than if we had tried to directly quantify those costs empirically, e.g., by comparing the productivity of colonies with more or less queen overproduction or manipulated brood sizes. In that sense, we believe that it would be a fruitful approach to model many other conflicts in the field of social evolution in more detail and achieve greater biological realism.

## Supplementary Material

qrad068_suppl_Supplementary_Material

## Data Availability

The raw data and R script used for the meta-analysis and the Mathematica notebook used to derive the ESS are publicly available under Dryad Repository (doi: 10.5061/dryad.1g1jwsv3d ).

## References

[CIT0001] Abbot, P., Abe, J., Alcock, J., Alizon, S., Alpedrinha, J. A. C., Andersson, M., Andre, J. -B., van Baalen, M., Balloux, F., Balshine, S., Barton, N., Beukeboom, L. W., Biernaskie, J. M., Bilde, T., Borgia, G., Breed, M., Brown, S., Bshary, R., Buckling, A., … Zink, A. (2011). Inclusive fitness theory and eusociality. Nature, 471(7339), E1–E4; author reply E9. 10.1038/nature09831 21430721 PMC3836173

[CIT0002] Alves, D. A., Menezes, C., Imperatriz-Fonseca, V. L., & Wenseleers, T. (2011). First discovery of a rare polygyne colony in the stingless bee *Melipona quadrifasciata* (Apidae, Meliponini). Apidologie, 42(2), 211–213. 10.1051/apido/2010053

[CIT0003] Avila, P., Priklopil, T., & Lehmann, L. (2021). Hamilton’s rule, gradual evolution, and the optimal (feedback) control of phenotypically plastic traits. Journal of Theoretical Biology, 526, 110602. 10.1016/j.jtbi.2021.110602 33508326

[CIT0004] Bego, L. R. (1983). On some aspects of bionomics in *Melipona bicolor bicolor* Lepeletier (Hymenoptera, Apidae, Meliponinae). Revista Brasileira de Entomologia, 27, 211–224.

[CIT0005] Bego, L. R. (1989). Behavioral interctions among queens of the polygynic stingless bee *Melipona bicolor bicolor* Lepeletier (Hymenoptera, Apidae). Brazilian Journal of Medical Biological Research, 22, 587–596.

[CIT0006] Bourke, A. F. G., & Ratnieks, F. L. W. (1999). Kin conflict over caste determination in social Hymenoptera. Behavioral Ecology and Sociobiology, 46(5), 287–297. 10.1007/s002650050622

[CIT0007] Brännström, A., & Sumpter, D. J. T. (2005). The role of competition and clustering in population dynamics. Proceedings of the Royal Society B: Biological Sciences, 272(1576), 2065–2072. 10.1098/rspb.2005.3185 PMC1559893 16191618

[CIT0008] Bueno, F. G. B., dos Santos, C. F., Otesbelgue, A., Menezes, C., van Veen, J., Blochtein, B., Gloag, R., Heard, T., Imperatriz-Fonseca, V. L., & Alves, D. A. (2023). The queens of the stingless bees: From egg to adult. Insectes Sociaux, 70(1), 43–57. 10.1007/s00040-022-00894-0

[CIT0009] Crozier, R. H., & Pamilo, P. (1996). Evolution of social insect colonies: Sex allocation and kin selection. Oxford University Press.

[CIT0010] Da Silva, D. L. N., Zucchi, R., & Kerr, W. E. (1972). Biological and behavioural aspects of the reproduction in some species of *Melipona* (Hymenoptera, Apidae, Meliponinae). Animal Behaviour, 20(1), 123–132. 10.1016/s0003-3472(72)80182-9 4664920

[CIT0011] Davies, N. G., & Gardner, A. (2018). Monogamy promotes altruistic sterility in insect societies. Royal Society Open Science, 5(5), 172190. 10.1098/rsos.172190 29892408 PMC5990772

[CIT0012] Day, T., & Taylor, P. D. (1997). Hamilton’s rule meets the Hamiltonian: Kin selection on dynamic characters. Proceedings of the Royal Society B: Biological Sciences, 264(1382), 639–644. 10.1098/rspb.1997.0090

[CIT0013] Dobata, S. (2012). Arms race between selfishness and policing: Two-trait quantitative genetic model for caste fate conflict in eusocial Hymenoptera. Evolution, 66(12), 3754–3764. 10.1111/j.1558-5646.2012.01745.x 23206134

[CIT0014] Engels, W., & Imperatriz-Fonseca, V. L. (1990). Caste development, reproductive strategies, and control of fertility in honey bees and stingless bees. Springer-Verlag.

[CIT0015] Frank, S. A. (1994). Kin selection and virulence in the evolution of protocells and parasites. Proceedings of the Royal Society B: Biological Sciences, 258(1352), 153–161. 10.1098/rspb.1994.0156 7838853

[CIT0016] Frank, S. A. (1995). Mutual policing and repression of competition in the evolution of cooperative groups. Nature, 377(6549), 520–522. 10.1038/377520a0 7566147

[CIT0017] Frank, S. A. (1998). Foundations of social evolution. Princeton University Press.

[CIT0018] Gardner, A., Alpedrinha, J., & West, S. A. (2012). Haplodiploidy and the evolution of eusociality: Split sex ratios. The American Naturalist, 179(2), 240–256. 10.1086/663683 22218313

[CIT0019] Gardner, A., & Úbeda, F. (2017). The meaning of intragenomic conflict. Nature Ecology & Evolution, 1(12), 1807–1815. 10.1038/s41559-017-0354-9 29109471

[CIT0020] Grüter, C. (2020). Stingless bees: Their behaviour, ecology and evolution. Springer Cham.

[CIT0021] Hartfelder, K., Makert, G. R., Judice, C. C., Pereira, G. A. G., Santana, W. C., Dallacqua, R., & Bitondi, M. M. G. (2006). Physiological and genetic mechanisms underlying caste development, reproduction and division of labor in stingless bees. Apidologie, 37, 144–163.

[CIT0022] Imperatriz-Fonseca, V. L., & Zucchi, R. (1995). Virgin queens in stingless bee (Apidae, Meliponinae) colonies: A review. Apidologie, 26(3), 231–244. 10.1051/apido:19950305

[CIT0023] Kennedy, P., Higginson, A. D., Radford, A. N., & Sumner, S. (2018). Altruism in a volatile world. Nature, 555(7696), 359–362. 10.1038/nature25965 29513655 PMC5986084

[CIT0024] Kennedy, P., Sumner, S., Botha, P., Welton, N. J., Higginson, A. D., & Radford, A. N. (2021). Diminishing returns drive altruists to help extended family. Nature Ecology & Evolution, 5(4), 468–479. 10.1038/s41559-020-01382-z 33589803 PMC7610556

[CIT0025] Kerr, W. E. (1950). Genetic determination of castes in the genus *Melipona*. Genetics, 35(2), 143–152. 10.1093/genetics/35.2.143 17247339 PMC1209477

[CIT0026] Koenig, W. D., Barve, S., Haydock, J., Dugdale, H. L., Oli, M. K., & Walters, E. L. (2023). Lifetime inclusive fitness effects of cooperative polygamy in the acorn woodpecker. Proceedings of the National Academy of Sciences of the United States of America, 120(19), e2219345120. 10.1073/pnas.2219345120 37126712 PMC10175847

[CIT0027] Kreider, J. J., Pen, I., & Kramer, B. H. (2021). Antagonistic pleiotropy and the evolution of extraordinary lifespans in eusocial organisms. Evolution Letters, 5(3), 178–186. 10.1002/evl3.230 34136267 PMC8190452

[CIT0028] Lehmann, L., Ravigné, V., & Keller, L. (2008). Population viscosity can promote the evolution of altruistic sterile helpers and eusociality. Proceedings of the Royal Society B: Biological Sciences, 275(1645), 1887–1895. 10.1098/rspb.2008.0276 PMC2593924 18460428

[CIT0029] Marshall, J. A. R. (2015). Social evolution and inclusive fitness theory: An introduction. Princeton University Press.

[CIT0030] Michener, C. D. (1974). The social behavior of the bees: A comparative study. Harvard University Press.

[CIT0031] Moo-Valle, H., Quezada-Euán, J. J. G., & Wenseleers, T. (2001). The effect of food reserves on the production of sexual offspring in the stingless bee *Melipona beecheii* (Apidae, Meliponini). Insectes Sociaux, 48(4), 398–403. 10.1007/pl00001797

[CIT0032] Nowak, M. A., Tarnita, C. E., & Wilson, E. O. (2010). The evolution of eusociality. Nature, 466(7310), 1057–1062. 10.1038/nature09205 20740005 PMC3279739

[CIT0033] Olejarz, J. W., Allen, B., Veller, C., & Nowak, M. A. (2015). The evolution of non-reproductive workers in insect colonies with haplodiploid genetics. Elife, 4, e08918. 10.7554/eLife.08918 26485033 PMC4755779

[CIT0034] Page, J. R. E., & Kerr, W. E. (1990). The evolution of monandry and queen replacement in *Melipona* (Hymenoptera: Apidae). Revista Brasileira de Genética, 13, 209–229.

[CIT0035] Parker, G. A., & Maynard Smith, J. (1990). Optimality theory in evolutionary biology. Nature, 348, 27–33.

[CIT0036] Quiñones, A. E., Henriques, G. J., & Pen, I. (2020). Queen–worker conflict can drive the evolution of social polymorphism and split sex ratios in facultatively eusocial life cycles. Evolution, 74, 15–28. 31520540 10.1111/evo.13844

[CIT0037] Quiñones, A. E., & Pen, I. (2017). A unified model of Hymenopteran preadaptations that trigger the evolutionary transition to eusociality. Nature Communications, 8, 15920. 10.1038/ncomms15920 PMC5490048 28643786

[CIT0038] Rasmussen, C., & Cameron, S. A. (2010). Global stingless bee phylogeny supports ancient divergence, vicariance, and long distance dispersal. Biological Journal of the Linnean Society, 99(1), 206–232. 10.1111/j.1095-8312.2009.01341.x

[CIT0039] Ratnieks, F. L. (2001). Heirs and spares: Caste conflict and excess queen production in *Melipona* bees. Behavioral Ecology and Sociobiology, 50(5), 467–473. 10.1007/s002650100388

[CIT0040] Reuter, M., & Keller, L. (2001). Sex ratio conflict and worker production in eusocial Hymenoptera. The American Naturalist, 158(2), 166–177. 10.1086/321311 18707345

[CIT0041] Ribeiro, M. F., Wenseleers, T., Santos Filho, P. S., & Alves, D. A. (2006). Miniature queens in stingless bees: Basic facts and evolutionary hypotheses. Apidologie, 37, 191–206.

[CIT0042] Santos-Filho, P. S., Alves, D. A., Eterovic, A., Imperatriz-Fonseca, V. L., & Kleinert, A. M. P. (2006). Numerical investment in sex and caste by stingless bees (Apidae: Meliponini): A comparative analysis. Apidologie, 37, 207–221.

[CIT0043] Shou, W., Bergstrom, C. T., Chakraborty, A. K., & Skinner, F. K. (2015). Theory, models and biology (p. e07158). eLife Sciences Publications, Ltd. 10.7554/eLife.07158 PMC4501050 26173204

[CIT0044] Sommeijer, M. J., de Bruijn, L. L. M., Meeuwsen, F. J. A. J., & Slaa, E. J. (2003). Reproductive behaviour of stingless bees: Nest departures of non-accepted gynes and nuptial flights in *Melipona favosa* (Hymenoptera: Apidae, Meliponini). Entomologische Berichten, 63, 7–13.

[CIT0045] Sommeijer, M. J., Koedam, D., & Monge, I. A. (1994). Social interactions of gynes and their longevity in queenright colonies of *Melipona favosa* (Apidae: Meliponinae). Netherlands Journal of Zoology, 45, 480–494.

[CIT0046] Stefan, A., Geritz, H., & Kisdi, E. (2012). Mathematical ecology: Why mechanistic models? Journal of Mathematical Biology, 65, 1411. 22159789 10.1007/s00285-011-0496-3

[CIT0047] Trivers, R. L., & Hare, H. (1976). Haploidploidy and the evolution of the social insect: The unusual traits of the social insects are uniquely explained by Hamilton’s kinship theory. Science, 191(4224), 249–263. 10.1126/science.1108197 1108197

[CIT0048] Van Oystaeyen, A., Alves, D. A., Oliveira, R. C., do Nascimento, D. L., do Nascimento, F. S., & Billen, J., & Wenseleers, T. (2013). Sneaky queens in *Melipona* bees selectively detect and infiltrate queenless colonies. Animal Behaviour, 86, 603–609.

[CIT0049] Wenseleers, T., Alves, D. A., Francoy, T. M., Billen, J., & Imperatriz-Fonseca, V. L. (2011). Intraspecific queen parasitism in a highly eusocial bee. Biology Letters, 7(2), 173–176. 10.1098/rsbl.2010.0819 20961883 PMC3061179

[CIT0050] Wenseleers, T., Gardner, A., & Foster, K. R. (2010). Social evolution theory: A review of methods and approaches. In T. Székely, A. J. Moore, & J. Komdeur (Eds.), Social behaviour: Genes, ecology and evolution (pp. 132–158). Cambridge University Press.

[CIT0051] Wenseleers, T., Hart, A. G., & Ratnieks, F. L. W. (2004a). When resistance is useless: Policing and the evolution of reproductive acquiescence in insect societies. The American Naturalist, 164(6), E154–E167. 10.1086/425223 29641925

[CIT0052] Wenseleers, T., Hart, A. G., Ratnieks, F. L. W., & Quezada‐Euán, J. J. G. (2004b). Queen execution and caste conflict in the stingless bee *Melipona beecheii*. Ethology, 110(9), 725–736. 10.1111/j.1439-0310.2004.01008.x

[CIT0053] Wenseleers, T., Helanterä, H., Alves, D. A., Duenez-Guzman, E., & Pamilo, P. (2013). Towards greater realism in inclusive fitness models: The case of worker reproduction in insect societies. Biology Letters, 9(6), 20130334. 10.1098/rsbl.2013.0334 24132088 PMC3871332

[CIT0054] Wenseleers, T., & Ratnieks, F. L. W. (2004). Tragedy of the commons in *Melipona* bees. Proceedings of the Royal Society B: Biological Sciences, 271(Suppl 5), S310–S312. 10.1098/rsbl.2003.0159 PMC1810046 15504003

[CIT0055] Wenseleers, T., Ratnieks, F. L. W., & Billen, J. (2003). Caste fate conflict in swarm‐founding social Hymenoptera: An inclusive fitness analysis. Journal of Evolutionary Biology, 16(4), 647–658. 10.1046/j.1420-9101.2003.00574.x 14632228

[CIT0056] Wenseleers, T., Ratnieks, F. L., Ribeiro, M. F., Alves, D. A., & Imperatriz-Fonseca, V. -L. (2005). Working-class royalty: Bees beat the caste system. Biology Letters, 1(2), 125–128. 10.1098/rsbl.2004.0281 17148145 PMC1626201

[CIT0057] West, S. (2009). Sex allocation. Princeton University Press.

[CIT0058] Weyna, A., Romiguier, J., & Mullon, C. (2021). Hybridization enables the fixation of selfish queen genotypes in eusocial colonies. Evolution Letters, 5(6), 582–594. 10.1002/evl3.253 34917398 PMC8645202

