# Supplementary material for: Toward greater realism in inclusive fitness models: the case of caste fate conflict in insect societies
Source: Evol Lett. 2024 Jan 11;8(3):387–96. doi: 10.1093/evlett/qrad068 (PMC11134464; doi:10.1093/evlett/qrad068)
Supplement: qrad068_suppl_Supplementary_Material [file qrad068_suppl_supplementary_material.zip › 2Supplementary_Material_Ferreira_et_al.._cleanVersion PDF.pdf]

Supplementary Material

Letter

Toward greater realism in inclusive fitness models: the case of caste fate conflict in insect societies

Helena Mendes Ferreira[1#], Denise Araujo Alves[2], Lloyd Cool[1,3], Cintia Akemi Oi[4], Ricardo Caliari Oliveira[5], Tom Wenseleers[1#]

[1]Laboratory of Socio-ecology and Social Evolution, Zoological Institute, University of Leuven, Leuven, Belgium

[2]Department of Entomology and Acarology, Luiz de Queiroz College of Agriculture, University of São Paulo, Piracicaba, Brazil

[3]Center of Microbial and Plant Genetics, University of Leuven, Leuven, Belgium

[4]Depatment of Genetics and Evolution, University College London, London, UK

[5]Departament de Biologia Animal, de Biologia Vegetal i d'Ecologia - Universitat Autònoma de Barcelona, 08193, Bellaterra (Barcelona), Spain

#Corresponding authors: HMF: helena.mendesferreira@kuleuven.be; TW: tom.wenseleers@kuleuven.be.

ORCID: 0000-0003-2090-4528 (HMF); 0000-0001-6525-427X (DAA); 0000-0001-9936-3124 (LC); 0000-13 0002-3682-8219 (CAO); 0000-0002-8996-1291 (RCO); 0000-0002-1434-861X (TW)

Short Running Title: Realistic models of caste conflict in insect societies

Keywords: Caste fate conflict, inclusive fitness theory, social evolution, stingless bees

Supplementary Methods

Data compilation

For our meta-analysis on levels of queen production in advanced eusocial bees, we focus on stingless bees as well as the honeybee, *Apis mellifera*, which is considered as an outgroup. In all these species, colony reproduction takes place through swarming, which means that one colony divides into two and the new one is headed by the mother queen (honeybees) or a newly reared daughter queen (stingless bees), accompanied by hundreds to thousands of workers. In total, based on a systematic search of published studies, conference abstracts, books and theses as well as some unpublished work, we found good quantitative data from a total of 43 species, which includes 21 *Melipona* stingless bees, 22 trigonine stingless bees, plus the honeybee, *Apis*

mellifera (see Table S5 for details). Our analysis includes all species for which accurate data on queen and worker brood production could be found (Table 2 & S5). To be able to test model predictions and calculate evolutionarily stable strategy levels (ESS) of queen overproduction, we also needed data on male parentage (proportion of adult males that are workers' sons as opposed to the queen's sons Ψ) (Table S1) as well as estimates of the percentage of queen laid eggs that were female (f) (Table S2), which could be recalculated into the average percentage of queen-laid eggs that are replaced by male worker-laid eggs following brood cell provisioning (w) (Table 2). These model parameters could be estimated for a total of 8 *Melipona* species. One species – *M. mondury* – for which some data on worker reproduction are available, was excluded from the analysis as it was the only *Melipona* species for which queen polyandry has been inferred (Viana *et al.* 2015), which would be highly atypical and still requires independent confirmation. To be able to calculate the colony level cost of queen overproduction for these 8 species, we further used data on worker life expectancies (L), which translate into expected daily worker mortality rates $\mu = 1/L$ (Table 2 & S32). For this, we made use of species-specific estimates where possible, but if these were not available, we used overall cross-species averages (Table 2& S3). Finally, the colony level cost of queen overproduction was also a function of per capita daily brood cell building rates (b) (cf. Model below). These have so far been measured only with lesser accuracy and for only few species. Hence, for this parameter we used the overall species average value for all 8 species (Table S4).

Evolutionarily stable strategy model

To formulate a more mechanistically motivated model of caste fate conflict in insect societies, we started from the biology of *Melipona* stingless bees, and explicitly derived the colony-level cost of queen production based on a dynamic model of colony growth and reproduction, i.e.

from a differential equation system that explicitly modelled the rate of producing new workers, swarms and males. The long-term net impact of queen overproduction on swarm and male production can then be integrated out and be expressed in function of underlying demographic parameters such as per capita cell building rates (b), worker life expectancy (L, inversely related to the worker mortality rate μ), the proportion of the queen's eggs that are female (f) and the probability with which the workers replace the queen's eggs with their own male eggs (w) (Table 1). To derive the ESS level of queen production, we use a neighbour-modulated fitness model (Frank 1998; Wenseleers *et al.* 2010), which is one approach to model kin conflict, but alternative formulations based on an actor-based inclusive fitness approach, a levels of selection approach or a plain population genetic model are also possible and would all give identical results under the given assumptions (cf. Ratnieks 2001; Wenseleers *et al.* 2003; Wenseleers *et al.* 2004a; Wenseleers *et al.* 2010). The possibility that some of the newly produced queens might be able to fly out of the colony and reproductively parasitise other nests is not explicitly considered, as this behaviour has only been well documented in *M. scutellaris* (Wenseleers *et al.* 2011; Van Oystaeyen *et al.* 2013) and *M. bicolor* (Reis *et al.* 2011) and anecdotally reported in only one other species, *M. favosa* (Sommeijer *et al.* 2003a; Sommeijer *et al.* 2003c), while in a third species – *M. beecheii* – queen parasitism was found to be absent (Oliveira *et al.* 2022), despite that species displaying high levels of queen production (Moo-Valle *et al.* 2001, Figure 3). Unlike the model of Wenseleers *et al.* (2013), we also do not consider the coevolutionary dynamics among several traits, but rather take the observed values of the parameters of our model as a given. All modelling was performed with the help of Wolfram Mathematica version 10.0 (Wolfram 1991).

Statistical analysis

All statistical analyses of the average percentage of females that develop into queens in *Melipona*, trigonine stingless bees, and honeybees, along with an analysis of the various individual parameters utilized that determine the ESS level of queen overproduction in *Melipona* species were conducted using R version 4.1.1 (R Core R Core Team 2021). The individual parameters that were used to calculate the ESS levels of queen production (b, L, ψ, f) as well as the observed % of females developing as queens (g) were calculated using generalized linear models (*glm* or *brglm2*) or generalized linear mixed models (*glmmPQL*) with appropriate error distribution and per-species and overall average means and 95% confidence intervals were calculated using the *emmeans* package. Firth's penalized generalized linear models with a Jeffrey's prior (*brglm2* package) were used to avoid complete separation when we were dealing with data that included proportions close to 0 or 1. In all models, species was added as a fixed effect and where possible, population, colony or brood comb were taken into account as nested random effects. Alternatively, any overdispersion was considered via the use of quasilikelihood models (for details see Tables S1-S5 and R script in supplementary material). To calculate the uncertainty on the per-species and overall overage predicted ESS level of queen production we resampled our parameters using the *mvrnorm* function from the *MASS* package, assuming they were multivariate normally distributed with expectation and variance given by the coefficient estimates and variance-covariance matrix of our fits. This uncertainty in the parameters was then propagated by entering the resampled parameters in the ESS formula (eqn. 6, main text), after which we calculated the 95% population prediction intervals as the 2.5% and 97.5% percentiles (for details see supplementary material).

Supplementary Figures and Tables

Table S1. Data on the percentage of males that are workers' sons (ψ) plus estimated 95% binomial confidence intervals in 8 *Melipona* species, together with the sample size (N_m: number of males for which the parentage was determined, N_a: effective number of males for which the parentage could be assigned given the power of the technique used, N: number of colonies). Method: method used to infer male parentage: allozyme markers (A), behavioural data (B), microsatellite markers (M). 95% confidence intervals on the percentage of adults males that are worker' sons were calculated using a Firth's penalized binomial GLM (*brglm2*) model fit in which species was coded as a fixed factor and the *emmeans* package. This method uses a Jeffreys invariant prior and a penalized maximum likelihood approach and was used to prevent complete separation caused by species where ψ is near 0 or near 1. All species have colonies that are either headed by a single queen or have an effective maternity close to 1 (in the case of *M. bicolor*, where a few egg laying queens sometimes co-occur).

**Species**	**Males that are workers' sons (ψ)** [95% CI]	N_m	N_a	N	**Method**	**References**
M. asilvai	19.9% [5.68-50.60%]	25	12	1	M	Paxton, unpubl. data (in Wenseleers *et al.* 2013)
M. beecheii	0.909% [0.055-13.20%]	108	54	13	M	Paxton *et al.* 2001
M. bicolor	37.5% [28.00-48.10%]	143	86.93	11	M	Alves, unpubl. data (in Wenseleers *et al.* 2013)
M. favosa	94.9% [92.90-96.40%]	604	604	4	B	Sommeijer *et al.* 1999
M. marginata	37.7% [19.90-59.70%]	41	20.5	3	M	Tóth *et al.* 2002
M. quadrifasciata	63.6% [43.00-80.20%]	47	23.5	2	M	Tóth *et al.* 2002
M. scutellaris	23.3% [19.30-27.70%]	622	388	50	M	Tóth *et al.* 2002; Alves *et al.* 2009b
M. subnitida	35.6% [31.20-40.10%]	592	442	20	B+A	Koedam *et al.* 1999; Contel & Kerr 1976; Koedam *et al.* 2005
Avg.	**33.75% [25.90-43.92%]**					

Table S2. Data on the percentage of queen-laid eggs that are female (f) in 8 *Melipona* species, together with the respective sample sizes used to obtain the estimates (N: number of colonies, B: number of brood cells, F: number of cells with female brood). 95% confidence intervals on the percentage of queen-laid eggs that are female were calculated using a binomial GLM and the *emmeans* package, using a model in which species was coded as a fixed factor. To obtain these estimates we analysed the proportion of cells with queen-laid eggs (total number of cells minus the number of cells times the inferred average proportion of males that were workers' sons in each species, Table S1) that were female as a binomial proportion.

**Species**	**Queen-laid eggs that are female (f)** [95% CI]	N	B	F	**References**
M. asilvai	88.1% [87.10-89.10%]	26	4,126	3,532	Gioli 2003; Santos-Filho *et al.* 2006
M. beecheii	79.4% [78.80-80.00%]	27	16,146	12,796	Darchen & Delage-Darchen 1975; van Veen & Arce 1999; Moo-Valle *et al.* 2001; Moo-Valle *et al.* 2004
M. bicolor	96.3% [96.00-96.50%]	24	23,299	21,929	Kerr 1950; Santos-Filho *et al.* 2006; Ferreira Jr *et al.* 2013
M. favosa	98.9% [98.80-99.10%]	81	21,878	18,087	Koedam 1999; Sommeijer *et al.* 2003b
M. marginata	92.5% [91.80-93.20%]	12	5,123	4,535	Kerr 1950; Hara 2001; Ribeiro & Kleinert, unpubl. data (in Wenseleers *et al.* 2013; Bueno *et al.* 2023)
M. quadrifasciata	97.4% [97.10-97.70%]	27	9,275	8,647	Kerr 1950; Bezerra 1995; Alves, unpubl. data (in Bueno *et al.* 2023)

M. scutellaris	88.2% [87.90-88.40%]	80	75,737	64,469	Pereira 2003; Alves 2010; Alves, unpubl. data (in Bueno *et al.* 2023)
M. subnitida	97.2% [96.70-97.70%]	33	4,936	4,727	Koedam *et al.* 2005 Santos-Filho *et al.* 2006
Avg.	**94.57% [94.37-94.70%]**				

Table S3. Data on the mean worker life expectancy (L) in 10 *Melipona* species, together with the sample sizes (n and N: number of individuals and colonies). 95% confidence intervals on average worker life expectancy were calculated using a log link gamma GLM and the *emmeans* package, using a model in which species was coded as a fixed factor. Assuming life expectancy is exponentially distributed the mean worker mortality rate per day (μ) was calculated as the reciprocal of worker life expectancy (i.e. $\mu = 1/L$).

**Species**	**Mean worker life expectancy *L* (days)** [95% CI]	***n***	***N***	**References**
M. beecheii	51.5 [49.4-53.6]	74	2	Biesmeijer & Tóth 1998; Wenseleers *et al.* 2004b
M. bicolor	44.0 [41.6-46.6]	50	1	Bego 1983
M. eburnea	39.5 [37.3-41.8]	101	2	Bustamante 2006
M. fasciculata	43.6 [41.9-45.4]	311	4	Giannini 1997[a]; Gomes *et al.* 2015
M. favosa	40.5 [38.9-42.2]	539	4	Roubik 1982; Sommeijer 1984
M. fulva	34.0 [32.1-36.0]	292	2	Roubik 1982
M. lateralis	44.5 [42.0-47.1]	92	2	Bustamante 2006
M. marginata	41.1 [38.8-43.5]	215	2	Mateus *et al.* 2019
M. scutellaris	43.8 [41.4-46.4]	431	2	Santos 2013
M. seminigra	30.0 [28.3-31.8]	15	1	Bustamante 2006
Avg.	**40.8 [39.8-41.9]**			

[a]*Melipona fasciculata* was orginally referred as *Melipona compressipes fasciculata* (Giannini 1997)

Table S4. Data on the reproductive efficiency b (the number of new brood cells built and provisioned per worker per day) in 4 *Melipona* species together with the respective sample sizes (N = total number of colonies). 95% confidence intervals were calculated using a log link quasipoisson GLM to consider overdispersion and the *emmeans* package with a model in which species was coded as a fixed factor. The log of the total number of worker days involved in building the brood cells (colony sizes (CS), *i.e.*, number of workers, times observation period in days) was included as a model offset to allow us to model the per capita rate at which new cells were built and provisioned. The expected marginal means obtained per species and the overall average were calculate by setting the offset to zero to return the average number of cells built per day per worker.

**Species**	**Reproductive efficiency *b*** [95% CI]	***N***	***CS***	**References**
M. beecheii	0.045 [0.038-0.053][a]	15	750-1839[d]	van Veen & Arce 1999; Wenseleers et al. 2013
M. bicolor	0.052 [0.032-0.085]	4	374	Müller, 1878; Alves, unpublished data
M. compressipes	0.045 [0.034-0.059][b]	8	85-1,410	Page & Kerr 1990
M. favosa	0.054 [0.037-0.077][c]	13	85-250	Sommeijer 1984; Sommeijer & De Bruijn 1984; Chinh *et al.* 2003[e]
Avg.	**0.049 [0.041-0.058]**			

[a-c] Estimated from total brood development times from egg to adult of [a]53, [b]50 and [c]51 days
[d] Colonies with atypical colony sizes excluded (smallest and largest colony sizes)
[e] Individual colony sizes linearly interpolated from average brood production of 11 brood cells/day (for details see supplemental Excel file)

Table S5. Data on the percentage of females developing as queens (*g*) and male production in 21 *Melipona* species, 22 trigonine stingless bee species and the honeybee *Apis mellifera*. The obtained values were determined from the inspection of brood combs and mention the respective sample sizes (*N*: number of colonies, *C*: number of combs, *B*: total number of brood cells). The mean percentage of females developing as queens (*g*) and 95% confidence intervals on were calculated using binomial GLM and GLMM models and the *emmeans* package. For queens reared only in worker cells (*Melipona*) we used a binomial Penalized Quasi-Likelihood *glmmPQL* model in which species was coded as a fixed effect and population, colony and comb were coded as nested random effects. For species that reared queens in both worker and royal cells (i.e. species with miniature queens) and species that reared queens in royal cells only we fit Firth's penalized binomial GLM models (*brglm2*) with a Jeffreys invariant prior to avoid complete separation due to the low counts, using a model in which either species (for queens produced in royal cells in species with miniature queens) or species plus colony nested in species were coded as fixed factors. When data was missing, we imputed missing values based on the average values calculated for all species overall. For details see supplemental R script.

Species	Queens in cell type	Queens reared in	% of females that develop as queens (*g*)	% males	*N*	*C*	*B*	References
Melipona asilvai	worker cells	worker cells only	7.22% [4.10-12.4%]	14.7% [9.49-22.0%]	26	159	4,126	Gioli 2003; Santos-Filho *et al.* 2006
M. beecheii	worker cells	worker cells only	11.2% [7.80-15.9%]	19.2% [16.4-22.3%]	27	149	16,146	van Veen & Arce 1999; Darchen & Delage-Darchen 1975; Moo-Valle *et al.* 2001; Moo-Valle *et al.* 2004
M. bicolor	worker cells	worker cells only	8.68% [5.30-13.9%]	6.53% [5.30-8.03%]	24	160	23,299	Kerr 1950; Santos-Filho *et al.* 2006; Ferreira Jr *et al.* 2013
M. colimana	worker cells	worker cells only	22.6% [8.48-47.8%]	21.4% [3.72-65.8%]	4	?	406	Macías-Macías & Quezada-Euán 2015
M. fasciculata[a]	worker cells	worker cells only	8.80% [4.80-15.6%]	11.9% [4.12-29.6%]	27	168	10,574	Morais *et al.* 2006; Leão 2019
M. favosa	worker cells	worker cells only	7.80% [4.32-13.7%]	17.1% [12.9-22.3%]	81	218	21,878	Koedam 1999; Sommeijer *et al.* 2003c
M. fuliginosa[b]	worker cells	worker cells only	6.38% [1.74-20.7%]	15.3% [4.02-43.7%]	1	2	196	Kerr 1950
M. fulva[c]	worker cells	worker cells only	15.7% [6.41-33.8%]	37.6% [13.4-70.1%]	2	?	472	Kerr *et al.* 1967
M. interrupta	worker cells	worker cells only	14.0% [5.21-32.6%]	13.25% [10.42-16.70%][h]	2	?	146	Kerr 1950
M. mandacaia	worker cells	worker cells only	13.0% [4.49-32.3%]	10.3% [1.60-45.1%]	2	?	445	Rodrigues *et al.* 2015
M. marginata	worker cells	worker cells only	10.1% [6.13-16.1%]	16.7% [11.8-23.1%]	12	?	5,123	Kerr 1950; Hara 2001; Ribeiro & Kleinert unpubl. data (in Wenseleers *et al.* 2013; Bueno *et al.* 2023)
M. melanoventer[d]	worker cells	worker cells only	8.67% [3.25-21.1%]	23.3% [9.79-45.8%]	2	4	524	Kerr 1950
M. mondury	worker cells	worker cells only	4.83% [2.24-10.1%]	4.56% [2.59-7.92%]	7	13	1,676	Lima 2004
M. orbignyi[e]	worker cells	worker cells only	7.50% [2.19-22.7%]	13.25% [10.42-16.70%][h]	2	2	166	Kerr 1950
M. quadrifasciata	worker cells	worker cells only	7.04% [4.33-11.3%]	6.74% [4.97-9.08%]	27	91	9,275	Kerr 1950; Bezerra 1995; Alves, unpubl. data (in Bueno *et al.* 2023)
M. quinquefasciata	worker cells	worker cells only	24.2% [9.89-48.1%]	13.25% [10.42-16.70%][h]	2	?	174	Kerr 1969
M. rufiventris	worker cells	worker cells only	6.35% [3.18-12.3%]	11.3% [8.11-15.6%]	11	32	4,642	Alves, unpubl. data (in Bueno *et al.* 2023)

M. scutellaris	worker cells	worker cells only	8.24% [6.03-11.2%]	12.4% [10.9-14.0%]	80	298	75,737	Pereira 2003; Alves 2010; Alves, unpubl. data (in Bueno *et al.* 2023)
M. seminigra	worker cells	worker cells only	17.6% [6.21-41.0%]	28.4% [5.51-73.0%]	1	?	364	Mateus *et al.* 2002
M. subnitida	worker cells	worker cells only	7.61% [4.59-12.3%]	5.66% [4.03-7.91%]	33	43	4,936	Koedam *et al.* 2005; Santos-Filho *et al.* 2006
M. trinitatis	worker cells	worker cells only	4.81% [1.63-13.4%]	7.76% [1.19-36.9%]	15	?	2,567	Sommeijer *et al.* 2003b
MELIPONA AVG.	worker cells	worker cells only	9.65% [8.07-11.50%]	13.25% [10.42-16.70%]				
Nannotrigona testaceicornis	worker cells	worker & royal cells	0.142% [0.0772-0.262%]	9.02% [2.76-25.8%]	15	114	20,154	Imperatriz-Fonseca *et al.* 1997; Cabral & Imperatriz-Fonseca 2008; Prato 2015; Wenseleers and Alves, unpubl. data (in Bueno *et al.* 2023)
Nannotrigona testaceicornis	royal cells	worker & royal cells	0.0287% [0.0124-0.0661%]	9.02% [2.76-25.8%]	15	114	20,154	Imperatriz-Fonseca *et al.* 1997; Cabral & Imperatriz-Fonseca 2008; Prato 2015; Wenseleers and Alves, unpubl. data (in Bueno *et al.* 2023)
Plebeia pugnax	worker cells	worker & royal cells	0.102% [0.0205-0.503%]	10.0% [1.53-44.2%]	2	2	1,911	Alves 2006; Wenseleers and Alves, unpubl. data (in Ribeiro *et al.* 2006)
Plebeia pugnax	royal cells	worker & royal cells	0.143% [0.0413-0.492%]	10.0% [1.53-44.2%]	2	2	1,911	Alves 2006; Wenseleers and Alves, unpubl. data (in Ribeiro *et al.* 2006)
Plebeia remota	worker cells	worker & royal cells	0.0386% [0.0122-0.122%]	14.1% [7.93-24.0%]	5	37	11,184	Imperatriz-Fonseca 1975; Ribeiro *et al.* 2003; Ribeiro *et al.* 2006; Alves *et al.* 2009a
Plebeia remota	royal cells	worker & royal cells	0.103% [0.0528-0.202%]	14.1% [7.93-24.0%]	5	37	11,184	Imperatriz-Fonseca 1975; Ribeiro *et al.* 2003; Ribeiro *et al.* 2006; Alves *et al.* 2009a
Scaptotrigona depilis	worker cells	worker & royal cells	0.0967% [0.0466-0.201%]	12.6% [6.27-23.7%]	>203[j]	>30[j]	12,0240	Lima *et al.* 2006; Koffler 2013; Alves, unpubl. data (in Bueno *et al.* 2023)
Scaptotrigona depilis	royal cells	worker & royal cells	0.0650% [0.0515-0.0819%]	12.6% [6.27-23.7%]	>203[j]	>30[j]	12,0240	Lima *et al.* 2006; Koffler 2013; Alves, unpubl. data (in Bueno *et al.* 2023)
Schwarziana quadripunctata	worker cells	worker & royal cells	0.577% [0.395-0.842%]	11.5% [7.74-16.6%]	18	96	14,578	Wenseleers *et al.* 2005; Santos-Filho *et al.* 2006
Schwarziana quadripunctata	royal cells	worker & royal cells	0.122% [0.0727-0.203%]	11.5% [7.74-16.6%]	18	96	14,578	Wenseleers *et al.* 2005; Santos-Filho *et al.* 2006
TRIGONINE STINGLESS BEES WITH MINIATURE QUEENS AVG.	worker cells	worker & royal cells	0.126% [0.081-0.196%]					
	royal cells	worker & royal cells	0.0803% [0.0568-0.114%]					
Apis mellifera	royal cells	royal cells only	0.0137% [0.00886-0.0211%]	-	?	?	?	Seeley 1985; Winston 1987
Friesella schrottkyi	royal cells	royal cells only	0.318% [0.112-0.905%]	3.17% [0.160-40.2%]	5	?	1,134	Sousa & Kleinert 2006
Frieseomelitta varia	royal cells	royal cells only	0.106% [0.0482-0.235%]	16.0% [3.64-48.9%]	3	?	6,976	Prato 2015
Heterotrigona moorei[f]	royal cells	royal cells only	0.483% [0.271-0.859%]	10.29% [6.23-16.55%][i]	1	10	2,652	Sakagami *et al.* 1989
Lepidotrigona flavibasis	royal cells	royal cells only	0.0634% [0.0429-0.0938%]	5.84% [0.809-32.0%]	24	?	220,494	Chinh 2004; Chinh & Sommeijer 2005
Partamona bilineata	royal cells	royal cells only	0.0418% [0.0166-0.105%]	10.29% [6.23-16.55%][i]	1	?	12,004	von Ihering 1903

Plebeia emerina	royal cells	royal cells only	0.0100% [0.000628-0.160%]	21.7% [1.55-83.0%]	4	16	6,358	Teixeira & Blochtein 2008
Plebeina hildebrandti	royal cells	royal cells only	0.425% [0.250-0.724%]	10.29% [6.23-16.55%][i]	1	?	3,538	Namu & Wittmann 2016
Scaptotrigona bipunctata	royal cells	royal cells only	0.0264% [0.0136-0.0511%]	14.4% [4.67-36.6%]	3	6	41,208	Alves, unpubl. data
Scaptotrigona postica	royal cells	royal cells only	0.0531% [0.0417-0.0675%]	3.16% [0.186-36.4%]	4	?	129,382	Bego 1977
Tetragona dorsalis	royal cells	royal cells only	0.00969% [0.00352-0.0267%]	10.29% [6.23-16.55%][i]	2	?	44,561	von Ihering 1903
Tetragonisca angustula	royal cells	royal cells only	0.0973% [0.0432-0.219%]	10.6% [3.91-25.8%]	6	72	15,454	Prato & Soares 2013; Prato 2015
Tetragonula carbonaria	royal cells	royal cells only	0.322% [0.219-0.475%]	12.5% [5.53-25.9%]	10	?	12,031	Gloag *et al.* 2007
Tetragonula laeviceps	royal cells	royal cells only	0.425% [0.169-1.07%]	10.29% [6.23-16.55%][i]	4	?	1,179	Chinh 2004
Trigona amalthea	royal cells	royal cells only	0.156% [0.106-0.228%]	16.9% [1.37-74.7%]	1	23	20,026	Schwarz 1948
Trigona crassipes	royal cells	royal cells only	0.0432% [0.0370-0.0505%]	10.29% [6.23-16.55%][i]	2	?	2,299,922	Camargo & Roubik 1991
Trigona hypogea	royal cells	royal cells only	0.00717% [0.00539-0.00953%]	10.29% [6.23-16.55%][i]	?	?	738,628	Camargo & Roubik 1991
Trigona spinipes[g]	royal cells	royal cells only	0.0633% [0.0183-0.218%]	10.29% [6.23-16.55%][i]	1	?	4,402	von Ihering 1903
TRIGONINE STINGLESS BEES AND HONEYBEES AVG.	royal cells	royal cells only	0.0695% [0.0555-0.0869%]	10.29% [6.23-16.55%]				

Species originally referred to as [a]*Melipona compressipes fasciculata*, [b]*Melipona flavipennis*, [c]*Melipona pseudocentris*, [d]*Melipona fasciata melanoventer*, [e]*Melipona favosa orbignyi*, [f]*Trigona moorei*, [g]*Trigona ruficrus*

[h]Missing values for individual species or accidentally recorded zero males; overall average % of males in the comb calculated across all *Melipona* species used instead

[i]Missing values for individual species; to estimate the number of workers in the brood, we used the overall average % of males in the combs observed across all trigonine stingless bee species

[j]Data on *N* and *C* from Lima *et al.* 2006

References

Alves, D.A. (2006). Queen dimorphism in *Plebeia pugnax* Moure (in litt.). In: *Anais do VII Encontro sobre Abelhas* Ribeirão Preto, p. 671.

Alves, D.A. (2010). Estratégias reprodutivas em Melipona, com ênfase em pequenas populações de *Melipona scutellaris* (Apidae, Meliponini) [PhD Thesis]. São Paulo, Universidade de São Paulo.

Alves, D.A., Imperatriz-Fonseca, V. & Santos-Filho, P. (2009a). Production of workers, queens and males in *Plebeia remota* colonies (Hymenoptera, Apidae, Meliponini), a stingless bee with reproductive diapause. *Genetic Molecular Research*, 8, 672-683.

Alves, D.A., Imperatriz-Fonseca, V.L., Francoy, T.M., Santos-Filho, P.S., Nogueira-Neto, P., Billen, J. *et al.* (2009b). The queen is dead—long live the workers: intraspecific parasitism by workers in the stingless bee *Melipona scutellaris*. *Molecular ecology*, 18, 4102-4111.

Bego, L.R. (1977). Aspectos da regulação social em *Nannotrigona (Scaptotrigona) postica* Latreille (Hymenoptera, Apidae, Meliponinae) [PhD Thesis]. Ribeirão Preto, University of São Paulo.

Bego, L.R. (1983). On some aspects of bionomics in *Melipona bicolor bicolor* Lepeletier (Hymenoptera, Apidae, Meliponinae). *Revista Brasileira de Entomologia*, 211-224.

Bezerra, J.M.D. (1995). Aspectos da reprodução de *Melipona quadrifasciata* (Hymenoptera, Apidae) [MSc Thesis]. Viçosa, University of Viçosa.

Biesmeijer, J.C. & Tóth, E. (1998). Individual foraging, activity level and longevity in the stingless bee *Melipona beecheii* in Costa Rica (Hymenoptera, Apidae, Meliponinae). *Insectes Sociaux*, 45, 427-443.

Bueno, F.G.B., dos Santos, C.F., Otesbelgue, A., Menezes, C., van Veen, J., Blochtein, B. *et al.* (2023). The queens of the stingless bees: from egg to adult. *Insectes Sociaux*, 70, 43-57.

Bustamante, N.C.R. (2006). Divisão de Trabalho em três aspécies de abelhas do gênero *Melipona* (Hymenoptera, Apidae) na Amazônia, Brasileira. [PhD Thesis]. Universidade Federal do Amazonas, Brazil.

Cabral, G.C.P. & Imperatriz-Fonseca, V.L. (2008). Baixa produção de rainhas em *Nannotrigona testaceicornis* durante a primavera e verão (Apidae, Meliponinae). . In: *Anais do VIII Encontro sobre Abelhas* Ribeirão Preto.

Camargo, J.M.F. & Roubik, D.W. (1991). Systematics and bionomics of the apoid obligate necrophages: the *Trigona hypogea* group (Hymenoptera: Apidae; Meliponinae). *Biological Journal of the Linnean Society*, 44, 13-39.

Chinh, T.X. (2004). Reproduction in eusocial bees (Apidae: Apini, Meliponini) [PhD Thesis]. Utrecht University.

Chinh, T.X., Grob, G.B., Meeuwsen, F.J. & Sommeijer, M.J. (2003). Patterns of male production in the stingless bee *Melipona favosa* (Apidae, Meliponini). *Apidologie*, 34, 161-170.

Chinh, T.X. & Sommeijer, M.J. (2005). Production of sexuals in the stingless bee *Trigona (Lepidotrigona) ventralis flavibasis* Cockerell (Apidae, Meliponini) in northern Vietnam. *Apidologie*, 36, 493-503.

Contel, E.P.B. & Kerr, W.E. (1976). Origin of males in *Melipona subnitida* estimated from data of an isozymic polymorphic system. *Genetica*, 46, 271-277.

Darchen, R. & Delage-Darchen, B. (1975). Contribution a l'Étude d'une Abeille du Mexique *Melipona Beecheii* B.(Hymenoptère: Apide). Le Déterminisme des Castes Chez les Mélipones. *Apidologie*, 6, 295-339.

Ferreira Jr, N.T., Blochtein, B. & Serrão, J.E. (2013). Seasonal production and spatial distribution of *Melipona bicolor schencki* (Apidae; Meliponini) castes in brood combs in southern Brazil. *Apidologie*, 44, 176-187.

Frank, S.A. (1998). *Foundations of social evolution*. Princeton University Press.

Giannini, K.M. (1997). Labor division in *Melipona compressipes fasciculata* Smith (Hymenoptera: Apidae: Meliponinae). *Anais da Sociedade Entomológica do Brasil*, 26, 153-162.
Gioli, L.D. (2003). Estudo dos aspectos reprodutivos relacionados à produção de machos em *Melipona asilvai* (Hymenoptera, Meliponinae) [MSc Thesis]. University of São Paulo, Ribeirão Preto.
Gloag, R.S., Beekman, M., Heard, T.A. & Oldroyd, B.P. (2007). No worker reproduction in the Australian stingless bee *Trigona carbonaria* Smith (Hymenoptera, Apidae). *Insectes Sociaux*, 54, 412-417.
Gomes, R.L.C., Menezes, C. & Contrera, F.A.L. (2015). Worker longevity in an Amazonian *Melipona* (Apidae, Meliponini) species: effects of season and age at foraging onset. *Apidologie*, 46, 133-143.
Hara, A. (2001). Influência da quantidade de alimento disponível na produção de indivíduos sexuais de *Melipona marginata* (Meliponini, Apidae) [Undergraduate Thesis]. São Paulo, Universidade de São Paulo.
Imperatriz-Fonseca, V.L. (1975). Notas sobre o comportamento de *Plebeia (Plebeia) remota* Holmberg (Apidae, Meliponinae). *Ciência e Cultura*, 27, 665-669.
Imperatriz-Fonseca, V.L., Cruz-Landim, C. & Moraes, R.L.M.S. (1997). Dwarf gynes in *Nannotrigona testaceicornis* (Apidae, Meliponinae, Trigonini). Behaviour, exocrine gland morphology and reproductive status. *Apidologie*, 28, 113-122.
Kerr, W.E. (1950). Genetic determination of castes in the genus *Melipona*. *Genetics*, 35, 143.
Kerr, W.E. (1969). Some aspects of the evolution of social bees (Apidae). *Journal of Evolutionary Biology*, 3, 119-175.
Kerr, W.E., Sakagami, S.F., Zucchi, R., Portugal-Araújo, V. & Camargo, J.M.F. (1967). Observações sobre a arquitetura dos ninhos e comportamento de algumas espécies de abelhas sem ferrão das vizinhanças de Manaus, Amazonas (Hymenoptera, Apoidea). In: *Atas do Simpósio sobre a biota Amazônica*, pp. 255-309.
Koedam, D. (1999). Production of queens, workers and males in the stingless bee *Melipona favosa* (Apidae: Meliponinae): patterns in time and space. *Netherlands Journal of Zoology*, 49, 289-302.
Koedam, D., Contrera, F.A.L., Fidalgo, A.O. & Imperatriz-Fonseca, V.L. (2005). How queen and workers share in male production in the stingless bee *Melipona subnitida* Ducke (Apidae, Meliponini). *Insectes Sociaux*, 52, 114-121.
Koedam, D., Contrera, F.A.L. & Imperatriz-Fonseca, V.L. (1999). Clustered male production by workers in the stingless bee *Melipona subnitida* Ducke (Apidae, Meliponinae). *Insectes Sociaux*, 46, 387-391.
Koffler, S. (2013). Influência da quantidade de recursos alimentares e da sazonalidade sobre a produção de sexuados em *Scaptotrigona aff. depilis* (Apidae, Meliponini) [MSc thesis Thesis]. Universidade de São Paulo.
Leão, K.L. (2019). Desenvolvimento colonial em abelhas nativas sem ferrão Amazônicas (Apidae: Meliponini): Tamanho populacional, nutrição e alocação fenotípica [PhD Thesis]. Federal University of Pará
Lima, F.V., Rech, A.R. & Balestieri, J.B.P. (2006). Frequência de castas em *Scaptotrigona depilis* (Moure, 1942) nos períodos de verão, outono, inverno e primavera. In: *Anais do VII Encontro sobre Abelhas* Ribeirão Preto.
Lima, M.A.P. (2004). Aspectos da biologia de *Melipona rufiventris* Lepeletier, 1836 e de *Melipona mondury* Smith, 1863 (Hymenoptera: Apidae, Meliponina) [MSc Thesis]. Federal University of Viçosa.
Macías-Macías, J.O. & Quezada-Euán, J.J.G. (2015). Stingless bees in a temperate climate: Oviposition behavior and duration of ontogenic development stages in *Melipona colimana* (Hymenoptera: Meliponini). *Journal of Apicultural Research*, 54, 255-259.

Mateus, S., Ferreira-Caliman, M.J., Menezes, C. & Grüter, C. (2019). Beyond temporal-polyethism: division of labor in the eusocial bee *Melipona marginata*. *Insectes Sociaux*, 66, 317-328.

Mateus, S., Hrncir, M., Faustino, C.D. & Zucchi, R. (2002). Ocorrência de intercastas adultas em colônias de *Melipona seminigra*: aspectos morfo-etologicos preliminares (Hymenoptera, Apidae, Meliponini). In: *Anais do V Encontro sobre Abelhas* Ribeirão Preto, pp. 211-216.

Moo-Valle, H., Quezada-Euán, J.J.G., Canto-Martín, J. & Gonzalez-Acereto, J.A. (2004). Caste ontogeny and the distribution of reproductive cells on the combs of *Melipona beecheii* (Apidae: Meliponini). *Apidologie*, 35, 587-594.

Moo-Valle, H., Quezada-Euán, J.J.G. & Wenseleers, T. (2001). The effect of food reserves on the production of sexual offspring in the stingless bee *Melipona beecheii* (Apidae, Meliponini). *Insectes Sociaux*, 48, 398-403.

Morais, M.M., Nascimento, F.S., Pereira, R.A. & Bego, L.R. (2006). Colony internal conditions related to caste production in *Melipona compressipes fasciculata* (Apidae, Meliponini). *Insectes Sociaux*, 53, 265-268.

Namu, F.N. & Wittmann, D. (2016). An African stingless bee *Plebeina hildebrandti* Friese nest size and design (Apidae, Meliponini. *African Journal of Ecology* 55, 111-114.

Oliveira, R.C., Di Pietro, V., Quezada-Euán, J.J.G., Pech, J.R., Moo-Valle, H. & Wenseleers, T. (2022). Tragedy of the commons in *Melipona* bees revisited. *Biology Letters*, 18, 20210498.

Page, J.R.E. & Kerr, W.E. (1990). The evolution of monandry and queen replacement in *Melipona* (Hymenoptera: Apidae). *Revista Brasileira de Genética*, 13, 209-229.

Paxton, R.J., Bego, L., Ruhnke, H., Ratnieks, F.W. & Quezada-Euán, J.J.G. (2001). Social evolution in stingless bees: Are the workers or is the queen in control of male production? *Advanced Ethology*, 236–237.

Pereira, R.A. (2003). Regulação social em *Melipona scutellaris*, com especial referência à reprodução e produtividade das colônias (Hymenoptera, Apinae, Meliponini) [MSc Thesis]. Ribeirão Preto, University of São Paulo.

Prato, M. (2015). Influência da quantidade de alimento sobre a produção de sexuados e a determinação de castas em três espécies de abelhas sem ferrão [PhD Thesis]. Ribeirão Preto, University of São Paulo.

Prato, M. & Soares, A.E.E. (2013). Production of sexuals and mating frequency in the stingless bee *Tetragonisca angustula* (Latreille)(Hymenoptera, Apidae). *Neotropical Entomology*, 42, 474-482.

R Core Team (2021). R: A Language and Environment for Statistical Computing R Foundation for Statistical Computing Vienna, Austria.

Ratnieks, F.L. (2001). Heirs and spares: caste conflict and excess queen production in *Melipona* bees. *Behavioral Ecology and Sociobiology*, 50, 467-473.

Reis, E.P.d., Campos, L.A.d.O. & Tavares, M.G. (2011). Prediction of social structure and genetic relatedness in colonies of the facultative polygynous stingless bee *Melipona bicolor* (Hymenoptera, Apidae). *Genetics and Molecular Biology*, 34, 338-344.

Ribeiro, M.F., Imperatriz-Fonseca, V.L. & Santos Filho, P.S. (2003). Exceptional high queen production in the Brazilian stingless bee *Plebeia remota*. *Studies on Neotropical Fauna Environment*, 38, 111-114.

Ribeiro, M.F., Wenseleers, T., Santos Filho, P.S. & Alves, D.A. (2006). Miniature queens in stingless bees: basic facts and evolutionary hypotheses. *Apidologie*, 37, 191-206.

Rodrigues, F., Ribeiro, M.F., Imperatriz-Fonseca, V.L. & Koedam, D. (2015). Brood production in *Melipona mandacaia* (Apidae, Meliponini). In: *Anais do XI Encontro sobre Abelhas* Ribeirão Preto.

Roubik, D.W. (1982). Seasonality in colony food storage, brood production and adult survivorship: studies of *Melipona* in tropical forest (Hymenoptera: Apidae). *Journal of the Kansas Entomological Society*, 55, 789-800.

Sakagami, S.F., Inoue, T., Yamane, S. & Salmah, S. (1989). Nests of the myrmecophilous stingless bee, *Trigona moorei*: how do bees initiate their nest within an arboreal ant nest? *Biotropica*, 21, 265-274.

Santos-Filho, P.S., Alves, D.A., Eterovic, A., Imperatriz-Fonseca, V.L. & Kleinert, A.M.P. (2006). Numerical investment in sex and caste by stingless bees (Apidae: Meliponini): a comparative analysis. *Apidologie*, 37, 207-221.

Santos, D.C.d.J. (2013). Divisão de trabalho e sua relação com a dinâmica dos hidrocarbonetos cuticulares em *Melipona scutellaris* (Hymenoptera, Apidae, Meliponini) [MSc Thesis]. Ribeirão Preto, University of São Paulo.

Schwarz, H.F. (1948). *Stingless bees (Meliponinae) of the western hemisphere*.

Seeley, T. (1985). *Honeybee ecology: a study of adaptation in social life*. Princeton University Press, Princeton, NJ.

Sommeijer, M.J. (1984). Distribution of labour among workers of *Melipona favosa* F.: age-polyethism and worker oviposition. *Insectes Sociaux*, 31, 171-184.

Sommeijer, M.J., Chinh, T.X. & Meeuwsen, F.J.A.J. (1999). Behavioural data on the production of males by workers in the stingless bee *Melipona favosa* (Apidae, Meliponinae). *Insectes Sociaux*, 46, 92-93.

Sommeijer, M.J. & De Bruijn, L.L.M. (1984). Social behaviour of stingless bees:" Bee-dances" by workers of the royal court and the rhythmicity of brood cell provisioning and oviposition behaviour. *Behaviour*, 89, 299-315.

Sommeijer, M.J., de Bruijn, L.L.M. & Meeuwsen, F.J.A.J. (2003a). Reproductive behaviour of stingless bees: solitary gynes of *Melipona favosa* (Hymenoptera: Apidae, Meliponini) can penetrate existing nests. *Entomologische Berichten*, 63, 31-35.

Sommeijer, M.J., De Bruijn, L.L.M., Meeuwsen, F.J.A.J. & Martens, E.P. (2003b). Natural patterns of caste and sex allocation in the stingless bees *Melipona favosa* and *M. trinitatis* related to worker behaviour. *Insectes Sociaux*, 50, 38-44.

Sommeijer, M.J., de Bruijn, L.L.M., Meeuwsen, F.J.A.J. & Slaa, E.J. (2003c). Reproductive behaviour of stingless bees: nest departures of non-accepted gynes and nuptial flights in *Melipona favosa* (Hymenoptera: Apidae, Meliponini). *Entomologische Berichten*, 63, 7-13.

Sousa, V.C. & Kleinert, A.M.P. (2006). Razão sexual em *Friesella schrottkyi* (Apidae, Meliponini): uma análise preliminar. In: *Anais do VII Encontro sobre Abelhas* Ribeirão Preto.

Teixeira, J.S.G. & Blochtein, B. (2008). Produção de operárias, machos e rainhas em colônias de *Plebeia emerina* (Friese, 1900) (Hymenoptera, Apidae, Meliponini). In: *Anais do VIII Encontro sobre Abelhas* Ribeirão Preto.

Tóth, E., Strassmann, J.E., Nogueira-Neto, P., Imperatriz-Fonseca, V.L. & Queller, D.C. (2002). Male production in stingless bees: variable outcomes of queen–worker conflict. *Molecular Ecology*, 11, 2661-2667.

Van Oystaeyen, A., Alves, D.A., Oliveira, R.C., do Nascimento, D.L., do Nascimento, F.S., Billen, J. *et al.* (2013). Sneaky queens in *Melipona* bees selectively detect and infiltrate queenless colonies. *Animal Behaviour*, 86, 603-609.

van Veen, J. & Arce, H.G.A. (1999). Nest and colony characteristics of log-hived *Melipona beecheii* (Apidae: Meliponinae). *Journal of Apicultural Research*, 38, 43-48.

Viana, M.V.C., de Carvalho, C.A.L., Sousa, H.A.C., Francisco, A.K. & Waldschmidt, A.M. (2015). Mating frequency and maternity of males in *Melipona mondury* (Hymenoptera: Apidae). *Insectes Sociaux*, 62, 491-495.

von Ihering, H. (1903). *Biologie der stachellosen Honigbienen Brasiliens*.

Wenseleers, T., Alves, D.A., Francoy, T.M., Billen, J. & Imperatriz-Fonseca, V.L. (2011). Intraspecific queen parasitism in a highly eusocial bee. *Biology Letters*, 7, 173-176.

Wenseleers, T., Gardner, A. & Foster, K.R. (2010). Social evolution theory: a review of methods and approaches. In: *Social behaviour: genes, ecology and evolution* (eds. Székely, T, Moore, AJ & Komdeur, J). Cambridge University Press, pp. 132-158.

Wenseleers, T., Hart, A.G. & Ratnieks, F.L.W. (2004a). When resistance is useless: policing and the evolution of reproductive acquiescence in insect societies. *The American Naturalist*, 164, E154-E167.
Wenseleers, T., Hart, A.G., Ratnieks, F.L.W. & Quezada-Euán, J.J.G. (2004b). Queen execution and caste conflict in the stingless bee *Melipona beecheii*. *Ethology*, 110, 725-736.
Wenseleers, T., Helanterä, H., Alves, D.A., Duenez-Guzman, E. & Pamilo, P. (2013). Towards greater realism in inclusive fitness models: the case of worker reproduction in insect societies. *Biology Letters*, 9, 20130334.
Wenseleers, T., Ratnieks, F.L., Ribeiro, M.d.F., Alves, D.d.A. & Imperatriz-Fonseca, V.-L. (2005). Working-class royalty: bees beat the caste system. *Biology Letters*, 1, 125-128.
Wenseleers, T., Ratnieks, F.L.W. & Billen, J. (2003). Caste fate conflict in swarm-founding social Hymenoptera: an inclusive fitness analysis. *Journal of Evolutionary Biology*, 16, 647-658.
Winston, M.L. (1987). *The biology of the honey bee*. Harvard University Press.
Wolfram, S. (1991). *Mathematica: a system for doing mathematics by computer*. Addison Wesley Longman Publishing Co., Inc.
