# Supplementary material for: Toward greater realism in inclusive fitness models: the case of caste fate conflict in insect societies
Source: Evol Lett. 2024 Jan 11;8(3):387–96. doi: 10.1093/evlett/qrad068 (PMC11134464; doi:10.1093/evlett/qrad068)

Supplementary *Mathematica* notebook

associated with manuscript
"Toward greater realism in inclusive fitness models: the case of caste fate conflict in insect societies" by Helena Mendes Ferreira, Denise Alves, Lloyd Cool, Cintia Akemi Oi, Ricardo Caliari Oliveira & Tom Wenseleers

---

PARAMETERS

t = time
W[t] = total number of workers at time t
M[t] = total number of males at time t
b = number of new cells provisioned per day per worker
f = proportion of the queen's eggs that are female
r = per capita growth rate in the size of workforce W[t]
s = colony size at which the colony splits in two and one new swarm is produced
Td = time it takes for the workforce to double in size (doubling time)
gij = probability that an individual focal larva j in a focal colony i develops as a queen
gi = average proportion of the female larvae that develop as queens in colony i
g = proportion of the female larvae that develop as queens on average in the whole population
g* = evolutionarily stable probability with which female larvae should develop as queens
w = average probability with which a queen-laid egg is replaced by a male worker-laid egg
μ = daily mortality rate of workers
ψ = proportion of males that are workers' sons = w/((1-w)*(1-f)+w)
cm = class reproductive value of males
cf = class reproductive value of females (queens)
cm/cf = relative class reproductive value of males vs. females (queens) = 1/(2-ψ) (Crozier & Pamilo 1996)
rm = regression relatedness to males reared in the colony
rf = regression relatedness to sister queens reared in the colony

EMPIRICAL PARAMETER ESTIMATES (cf. R script)

Average parameter estimates across 8 *Melipona* species with best parameters estimates :

```
bavg = 0.04863397; blower = 0.04090696; bupper = 0.05782054;
μavg = 1 / 40.83532; μlower = 1 / 41.94643; μupper = 1 / 39.75364;
favg = 0.9457439; flower = 0.9437444; fupper = 0.9476763;
ψavg = 0.3375385; ψlower = 0.248973; ψupper = 0.4391854;
wavg = 0.0262; wlower = 0.0006; wupper = 0.1894; (* cf Table S5 *)
```

```
wfromψ[ψ_, f_] = Solve[ψ == w / ((1 - w) * (1 - f) + w), w][[1]][[1]][[2]]
```

$$\frac{(-1+f)\,\psi}{-1+f\,\psi}$$

Parameter estimates for *Melipona favosa*, a species where most males are worker produced (ψ=0.95) and f co-evolved to a very high value (f=0.99) (cf. Wenseleers *et al. Biol. Lett.* 2013) :

```
bfavosa = 0.05353166;
bfavosalower = 0.03707310;
bfavosaupper = 0.07729697; (* cf ess_table *)
μfavosa = 1 / 40.50000; μfavosalower = 1 / 43.22473;
μfavosaupper = 1 / 37.94703; (* cf df_worker_life_expectancy_melipona *)
ffavosa = 0.9894761; ffavosalower = 0.9878891;
ffavosaupper = 0.9908571;
ψfavosa = 0.949256198;
ψfavosalower = 0.9286093666;
ψfavosaupper = 0.9641622;
gfavosa = 0.07801616; gfavosalower = 0.04319330;
gfavosaupper = 0.1368967; (* queen production *)
wfavosa = 0.1639; wfavosalower = 0.1154; wfavosaupper = 0.2273; (* cf Table S5 *)
```

Parameter estimates for *Melipona beecheii*, a species where all males are queen produced (ψ=0) and f co-evolved to a lower value (f=0.79) (cf. Wenseleers *et al. Biol. Lett.* 2013) :

```
bbeecheii = 0.04508358;
bbeecheiilower = 0.03848672;
bbeecheiiupper = 0.05281117;
μbeecheii = 1 / 51.45000;
μbeecheiilower = 1 / 54.91142;
μbeecheiiupper = 1 / 48.20678;
fbeecheii = 0.7940159; fbeecheiilower = 0.7877019;
fbeecheiiupper = 0.8001898;
ψbeecheii = 0; ψbeecheiilower = 0; ψbeecheiiupper = 0.1322522;
gbeecheii = 0.11231784; gbeecheiilower = 0.07799982;
gbeecheiiupper = 0.1591290; (* queen production *)
wbeecheii = 0.0019; wbeecheiilower = 0.0001;
wbeecheiiupper = 0.0344; (* cf Table S5 *)
```

RELATIVE RATE OF SWARM PRODUCTION IN FUNCTION OF QUEEN OVERPRODUCTION

If b is the per-capita rate with which workers build and provision new cells, f is the proportion of those cells that contain female eggs, gi is the proportion of the female larvae that develop as

queens rather than workers in colony *i* and *μ* is the daily worker mortality rate, it is clear that the worker population will grow at a rate τWi = (b*f*(1-w)*(1-gi)-*μ*), i.e. W'[t]=τWi*W[t] (see also model in Bulmer 1994; note: Page & Kerr 1990 have indeed shown that in *Melipona compressipes* there is a linear relationship between the number of workers and the number of brood present.

From this, the number of workers after *t* days is (we assume the colony was swarm founded and had an initial size of *s*/2 (W[0]==s/2)) :

```
Wt = DSolve[{W'[t] == (b * f * (1 - w) * (1 - gi) - μ) * W[t],
      (* net growth rate τWi = b*f*(1-w)*(1-gi)-μ *)
          W[0] == s / 2}, W[t], t][[1]][[1]][[2]]
```

$$\frac{1}{2} e^{t\,(b\,f-b\,f\,gi-b\,f\,w+b\,f\,gi\,w-\mu)}\,s$$

```
WT = DSolve[{W'[t] == τWi * W[t], (* net growth rate τWi = b*f*(1-w)*(1-gi)-μ *)
          W[0] == s / 2}, W[t], t][[1]][[1]][[2]]
```

$$\frac{1}{2} e^{t\,\tau Wi}\,s$$

The number of days *Td* it takes before the colony will split in two and produce a new swarm (i.e. the doubling time) is log(2) / τWi = log(2) / (b*f*(1-w)*(1-gi)-*μ*) :

```
Td[gi_, b_, f_, μ_, w_] = FullSimplify[Solve[Wt == s, t, Reals][[1]][[1]][[2]][[1]]]
```

$$\frac{\mathrm{Log}[2]}{b\,f\,(-1+gi)\,(-1+w)-\mu}$$

Hence, the rate of swarm production *S* as a function of the level of queen overproduction *gi* is 1 / Td :

```
S[gi_, b_, f_, μ_, w_] = FullSimplify[(1 / Td[gi, b, f, μ, w])]
```

$$\frac{b\,f\,(-1+gi)\,(-1+w)-\mu}{\mathrm{Log}[2]}$$

This would drop to zero when *gi* is larger than

```
gicrit[b_, f_, μ_, w_] =
 FullSimplify[Solve[S[gi, b, f, μ, w] == 0, gi][[1]][[1]][[2]]]
```

$$1+\frac{\mu}{b\,f\,(-1+w)}$$

i.e. when the rate of producing new workers *(b*f*(1-w)*(1-gi))* would be less than the rate at which they die (*μ*).

For empirically estimated parameter values this occurs when 45% of all female larvae would develop as queens:

```
gicrit[bavg, favg, μavg, wavg]
```

0.45326

```
gicrit[bfavosa, ffavosa, μfavosa, wfavosa]
```

0.442467

```
gicrit[bbeecheii, fbeecheii, μbeecheii, wbeecheii]
```

0.456007

```
Plot[{ gicrit[bbeecheii, fbeecheii, 1 / 1, 0],
  (* for param values of Melipona beecheii, red *)
     gicrit[bfavosa, ffavosa, 1 / 1, wfromψ[ψfavosa, ffavosa]]},
 (* for param values of Melipona favosa, blue *)
 {1, 30, 100}, Frame → True,
 FrameLabel → {"Worker life expectancy (days)", "Critical gi value"},
 PlotStyle -> {Red, Blue}
]
```

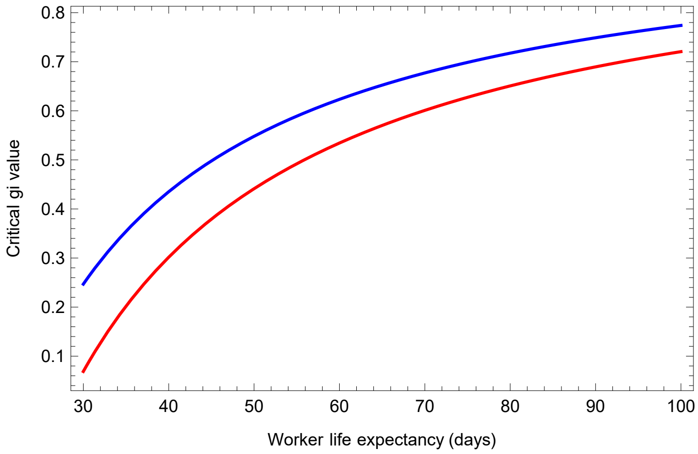

Hence, relative swarm production of a focal colony *i* where a proportion *gi* of all female larvae become queens vs that of other colonies in the population where a proportion *g* of all female larvae become queens is

```
Ws[gi_, g_, b_, f_, μ_, w_] = S[gi, b, f, μ, w] / S[g, b, f, μ, w]
```

$$\frac{b\, f\, (-1 + gi)\, (-1 + w) - \mu}{b\, f\, (-1 + g)\, (-1 + w) - \mu}$$

The mean relative probability that a focal female larva *j* in colony *i* who becomes a queen with probability *gij* will be able to inherit a swarm is therefore

```
Wf = Ws[gi, g, b, f, μ, w] * (gij / gi)
```

$$\frac{gij\, (b\, f\, (-1 + gi)\, (-1 + w) - \mu)}{gi\, (b\, f\, (-1 + g)\, (-1 + w) - \mu)}$$

We can look at the cost a given level of queen overproduction *gi* will confer by comparing the rate of swarm production in a colony where a proportion *gi* of all females develop as queens relative to colonies where queens would not be overproduced (*g*=0). In the absence of worker mortality (μ=0) or with very high rates of brood cell construction (b→∞), the cost function would reduce to that assumed in the original models on *Melipona* caste fate conflict (Ratnieks 2001, Wenseleers et al. 2003) (grey line), with swarm production only reducing to zero when all females would develop as queens. However, for realistic parameter values as estimated for *M. beecheii* (which has no worker reproduction, *w*=0, but where queens lay a relatively large amount of male eggs, *f*=0.77) (red line) or *M. favosa* (where most males are workers' sons, ψ=0.945, *w*=0.16, but with queens laying mostly female eggs, *f*=0.99) (blue line), swarm production would already reduce to zero when ca. half of all female larvae would develop as queens, and the colony-level cost would also be relatively more severe if most of the males would be workers' sons (blue line). From this, we would expect the ESS level of queen production to be ca. half of that from the original models of Ratnieks (2001) and Wenseleers et al. (2003).

```
Plot[{Ws[gi, 0, bavg, favg, 0, wfromψ[ψavg, favg]],
   (* cost function with zero worker mortality  *)
  Ws[gi, 0, bavg, favg, μavg, wfromψ[ψavg, favg]],
   (* for avg param values, black *)
  Ws[gi, 0, bavg, fbeecheii, μavg, wfromψ[ψbeecheii, ffavosa]],
   (* for param values of Melipona beecheii, red *)
  Ws[gi, 0, bavg, ffavosa, μavg, wfromψ[ψfavosa, ffavosa]]},
 (* for param values of Melipona favosa, blue *)
 {gi, 0, 1}, Frame → True, FrameLabel →
  {"Prop. of females becoming queens (gi)", "Relative swarm production (Ws)"},
 PlotStyle -> {Gray, Black, Red, Blue}]
```

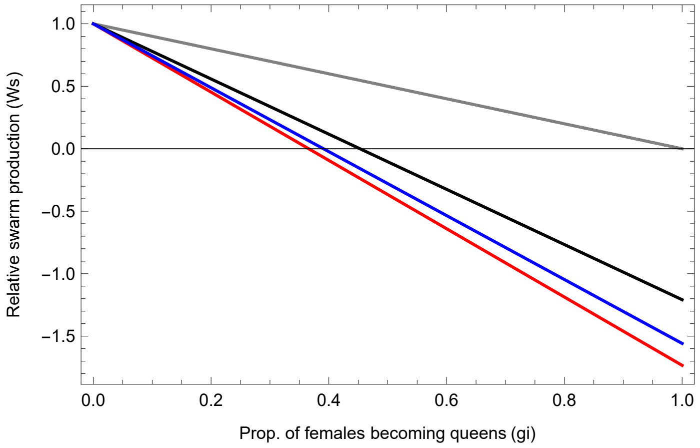

Note: if there was no worker mortality (i.e. workers would have an infinite lifespan) or if workers built a very large number of cells per day per worker (b→∞), this relative swarm production would reduce to that assumed in Ratnieks (2001) and Wenseleers et al. (JEB 2003), but this would evidently be highly unrealistic :

```
Ws[gi, g, b, f, 0, w]
```

$$\frac{-1+gi}{-1+g}$$

```
Limit[Ws[gi, g, b, f, μ, w], b → ∞]
```

$$\frac{-1+gi}{-1+g}$$

RELATIVE MALE PRODUCTION IN FUNCTION OF QUEEN OVERPRODUCTION

Similarly as above, it can be seen that the number of new males produced per day is M'[t]=b*(w+(1-w)*(1-f))*W[t], which implies that the total number of males produced *M* from time=0 up to the doubling time *Td* in the population would be

$$M[gi\_, g\_, b\_, f\_, \mu\_, w\_, s\_] = \int_0^{Td[g,b,f,\mu,w]} (b * (w + (1 - w) * (1 - f)) * Wt)\, dt$$

$$\left(\left(-1+2^{\frac{b\,f\,(-1+gi)\,(-1+w)-\mu}{b\,f\,(-1+g)\,(-1+w)-\mu}}\right) b\,s\,(1+f\,(-1+w))\right) \Big/ \left(2\,(b\,f\,(-1+gi)\,(-1+w)-\mu)\right)$$

In simplified notation M=

$$\int_0^{Td} \tau M * WT \, dt$$

$$\frac{\left(-1 + e^{Td\, \tau Wi}\right) s\, \tau M}{2\, \tau Wi}$$

Hence, over this period, the relative rate at which new males are produced in a colony where a proportion *gi* of all females develop as queens compared to the wild type where a prop. *g* develop as queens is

```
Wm[gi_, g_, b_, f_, μ_, w_] =
 FullSimplify[M[gi, g, b, f, μ, w, s] / M[g, g, b, f, μ, w, s]]
```

$$\left(\left(-1 + 2^{\frac{b f (-1+gi) (-1+w)-\mu}{b f (-1+g) (-1+w)-\mu}}\right) (b f (-1 + g) (-1 + w) - \mu)\right) \Big/ (b f (-1 + gi) (-1 + w) - \mu)$$

In simplified notation Wm =

```
FullSimplify[
 ((-1 + e^(Td τWi)) s τM / (2 τWi) /. {Td → Log[2] / τW}) / ((-1 + e^(Td τW)) s τM / (2 τW) /. {Td → Log[2] / τW})]
```

$$\frac{\left(-1 + 2^{\frac{\tau Wi}{\tau W}}\right) \tau W}{\tau Wi}$$

We can look at the cost a given level of queen overproduction *gi* will confer by comparing the rate of male production in a colony where a proportion *gi* of all females develop as queens relative to colonies where queens would not be overproduced (*g*=0). Here, even in the absence of worker mortality (μ=0) or with very high rates of brood cell construction (b→∞), relative male production would not reduce to that assumed in the original models on *Melipona* caste fate conflict (Ratnieks 2001, Wenseleers et al. 2003) (grey line), as relative male production would drop to log(2) rather than 0 if all females would develop as queens. This is because if a colony swarms, the original workers that found the colony will still end up raising new males, even if all newly produced female larvae would develop as queens and would not contribute to any growth of the work force. For realistic parameter values as estimated for *M. beecheii* (which has no worker reproduction, *w*=0, but where queens lay a relatively large amount of male eggs, *f*=0.77) (red line) or *M. favosa* (where most males are workers' sons, ψ=0.945, *w*=0.16, but with queens laying mostly female eggs, *f*=0.99) (blue line), relative male production, drops to a lower level if all females would develop as queens, but not to zero as in the assumed 1-g cost function in Ratnieks (2001) and Wenseleers et al. (2003). In addition, the cost function is convex rather than linear. Finally, the colony-level cost can be seen to be relatively more severe if most males would be workers' sons (blue line).

```
Plot[{Wm[gi, 0, bavg, favg, 0, wfromψ[ψavg, favg]],
  (* cost function with zero worker mortality  *)
  Wm[gi, 0, bavg, favg, μavg, wfromψ[ψavg, favg]],
  (* for avg param values, black *)
  Wm[gi, 0, bavg, fbeecheii, μavg, wfromψ[ψbeecheii, fbeecheii]],
  (* for param values of Melipona beecheii, red *)
  Wm[gi, 0, bavg, ffavosa, μavg, wfromψ[ψfavosa, ffavosa]]},
 (* for param values of Melipona favosa, blue *)
 {gi, 0, 1}, Frame → True, FrameLabel →
  {"Prop. of females becoming queens g", "Relative male production"},
 PlotRange → {0, 1},
 PlotStyle -> {Green, Black, Red, Blue}]
```

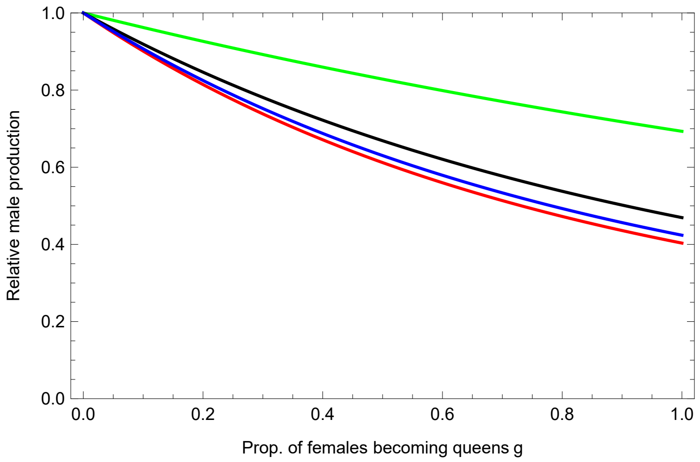

For *gi* approaching 1 we would get these values for relative male production :

```
Wm[0.999, 0, bavg, favg, μavg, wfromψ[ψavg, favg]]
```

0.469767

```
Wm[0.999, 0, bavg, fbeecheii, μavg, wfromψ[ψbeecheii, fbeecheii]]
```

0.403711

```
Wm[0.999, 0, bavg, ffavosa, μavg, wfromψ[ψfavosa, ffavosa]]
```

0.424174

```
N[Log[2]]
```

0.693147

Note: if there was no worker mortality (μ = 0, i.e. workers would have an infinite lifespan) or if workers built a very large number of cells per day per worker (b → ∞), this relative male production would reduce to log[2], as opposed to zero as assumed in Ratnieks (2001) and Wenseleers et al. (JEB 2003), but this would evidently be highly unrealistic :

```
Limit[Wm[gi, 0, b, f, 0, w], gi → 1]
```

Log[2]

```
Limit[Limit[Wm[gi, 0, b, f, μ, w], b → ∞], gi → 1]
```

Log[2]

SELECTION DIFFERENTIAL FOR QUEEN PRODUCTION

With these newly derived functions for *Wf* and *Wm*, it is straighforward to calculate the selection differential σ on the probability with which females should develop as queens *g*. In particular, from kin selection theory, using a neighbour-modulated fitness framework, and assuming that many female larvae within a given colony compete to become queens, this selection differential can be seen to be (cf. Wenseleers et al. 2003 & Wenseleers et al. 2010) :
σ=cf*D[Wf,gij]*1+cf*D[Wf,gi]*rf+cm*D[Wm,gi]*rm
which is proportional to
σ=D[Wf,gij]*1+D[Wf,gi]*rf+(cm/cf)*D[Wm,gi]*rm

```
Clear[rf, rm]
```

```
σ = D[Wf, gij] * 1 + D[Wf, gi] * rf + cmovercf * D[Wm[gi, g, b, f, μ, w], gi] * rm
```

$$rf\left(\frac{b\,f\,gij\,(-1+w)}{gi\,(b\,f\,(-1+g)\,(-1+w)-\mu)}-\frac{gij\,(b\,f\,(-1+gi)\,(-1+w)-\mu)}{gi^2\,(b\,f\,(-1+g)\,(-1+w)-\mu)}\right)+\frac{b\,f\,(-1+gi)\,(-1+w)-\mu}{gi\,(b\,f\,(-1+g)\,(-1+w)-\mu)}+cmovercf\;rm\left(-\left(\left(\left(-1+2^{\frac{b\,f\,(-1+gi)\,(-1+w)-\mu}{b\,f\,(-1+g)\,(-1+w)-\mu}}\right)b\,f\,(-1+w)\,(b\,f\,(-1+g)\,(-1+w)-\mu)\right)\Big/(b\,f\,(-1+gi)\,(-1+w)-\mu)^2\right)+\left(2^{\frac{b\,f\,(-1+gi)\,(-1+w)-\mu}{b\,f\,(-1+g)\,(-1+w)-\mu}}\,b\,f\,(-1+w)\,Log[2]\right)\Big/(b\,f\,(-1+gi)\,(-1+w)-\mu)\right)$$

which in the limit of weak selection (gij and gi approx. being equal to g) becomes

```
σ = σ /. {gij → g, gi → g}
```

$$\frac{1}{g}+rf\left(-\frac{1}{g}+\frac{b\,f\,(-1+w)}{b\,f\,(-1+g)\,(-1+w)-\mu}\right)+cmovercf\;rm\left(-\frac{b\,f\,(-1+w)}{b\,f\,(-1+g)\,(-1+w)-\mu}+\frac{2\,b\,f\,(-1+w)\,Log[2]}{b\,f\,(-1+g)\,(-1+w)-\mu}\right)$$

```
D[Wf, gij] /. {gi → g}
```

$$\frac{1}{g}$$

```
D[Wf, gi] /. {gij → g, gi → g}
```

$$-\frac{1}{g}+\frac{b\,f\,(-1+w)}{b\,f\,(-1+g)\,(-1+w)-\mu}$$

```
D[Wm[gi, g, b, f, μ, w], gi] /. {gi → g}
```

$$-\frac{b\,f\,(-1+w)}{b\,f\,(-1+g)\,(-1+w)-\mu}+\frac{2\,b\,f\,(-1+w)\,Log[2]}{b\,f\,(-1+g)\,(-1+w)-\mu}$$

The ESS (evolutionarily stable strategy) will then be reached when this selection differential is zero, which will be reached when larvae develop as queens with a probability g* of

```
ess = FullSimplify[Solve[σ == 0, g][[1]][[1]][[2]]]
-(((-1 + rf) (b f (-1 + w) + μ)) / (b f (-1 + w) (1 + cmovercf rm (-1 + Log[4]))))
```

with signs in more logical way:

```
((1 - rf) (b f (1 - w) - μ)) / (b f (1 - w) (1 - cmovercf rm (1 - Log[4])))
```

Note that the proportion of males in the population that will be produced by the workers *ψ* is

```
ψ[w_, f_] = w / ((1 - w) * (1 - f) + w);
```

Hence, the regression relatedness of female larvae to new queens and to males produced by the colony rf and rm is given by (note that rbrothers = 1/2 and rnephews = rsisters) :

```
rf = rsisters;
rm [w_, f_] = (rbrothers * (1 - ψ[w, f]) + rnephews * ψ[w, f]) /.
   {rbrothers → 1 / 2, rnephews → rsisters};
```

Due to haploidy, the relative class reproductive value of males vs. queens is given by

```
cmovercf[w_, f_] = 1 / (2 - ψ[w, f]);
(* relative class reproductive value of males vs queens,
cf. Crozier & Pamilo 1996,
https://books.google.be/books?id=HUsgAQAAMAAJ&q=pamilo&dq=
   pamilo&hl=en&sa=X&ved=2ahUKEwjA06LHyPT7AhWQTcAKHV7FDdUQ6AF6BAgDEAI *)
```

```
ESS[rsisters_, rbrothers_, b_, f_, μ_, w_] =
 ess /. {cmovercf -> cmovercf[w, f], rm → rm[w, f]} /. {rnephews → rsisters}
```

$$-\left(\left((-1+\text{rsisters})\ (b\ f\ (-1+w)+\mu)\right)\Big/\left(b\ f\ (-1+w)\left(1+\left(\left(\frac{\text{rsisters}\ w}{(1-f)\ (1-w)+w}+\frac{1}{2}\left(1-\frac{w}{(1-f)\ (1-w)+w}\right)\right)(-1+\text{Log}[4])\right)\Big/\left(2-\frac{w}{(1-f)\ (1-w)+w}\right)\right)\right)\right)$$

where $w(\psi,f)=\frac{(1-f)\,\psi}{1-f\,\psi}$

```
wfromψ[ψ_, f_] = Solve[ψ == w / ((1 - w) * (1 - f) + w), w][[1]][[1]][[2]]
```

$$\frac{(-1+f)\ \psi}{-1+f\ \psi}$$

PREDICTED ESS IN ABSENCE OF WORKER MORTALITY OR UNDER HIGH PER CAPITA RATE OF BROOD CELL CONSTRUCTION (UNREALISTIC)

If workers lived forever (if their mortality rate was zero, i.e. μ = 0) or if workers could build a very large number of cells per day (b→∞) this ESS would be close to that found in the original models of Ratnieks (2001) and Wenseleers et al. 2003 :

E.g. in the absence of worker reproduction (w = 0) :

```
FullSimplify[ESS[rsisters, rbrothers, b, f, 0, 0], w > 0 && w < 1 && f > 0 && f < 1]
```

$$\frac{2-2\,\text{rsisters}}{2+\text{rbrothers}\,(-1+\text{Log}[4])}$$

```
FullSimplify[ESS[3 / 4, 1 / 2, b, f, 0, 0], w > 0 && w < 1 && f > 0 && f < 1]
```

$$\frac{1}{3+\text{Log}[4]}$$

```
N[FullSimplify[ESS[3 / 4, 1 / 2, b, f, 0, 0], w > 0 && w < 1 && f > 0 && f < 1]]
(* original prediction was 20% *)
```

0.227983

```
Plot[{100 * ESS[0.75, 0.5, bavg, favg, 1 / 1000, wfromψ[ψavg, favg]],
   100 * ESS[0.75, 0.5, bavg, fbeecheii, 1 / 1000, wfromψ[ψbeecheii, fbeecheii]],
  100 * ESS[0.75, 0.5, bavg, ffavosa, 1 / 1000, wfromψ[ψfavosa, ffavosa]]},
 {b, 0.03, 0.1}, AxesOrigin → {0.03, 0}, Frame → True,
 FrameLabel → {"Nr of new cells produced per day per worker",
   "ESS queen production (%)"},
 PlotStyle -> { Black, Red, Blue}] (* only slightly different
 because of difference in cost function for males *)
```

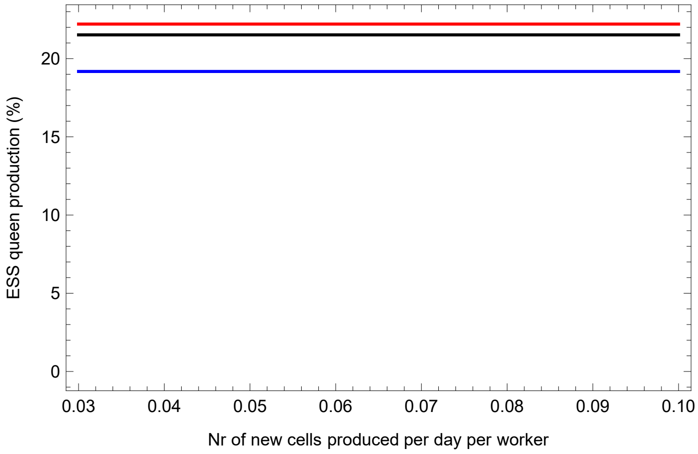

PREDICTED ESS VALUES

```
ESS[0.75, 0.5, bavg, fbeecheii, μbeecheii, wfromψ[ψbeecheii, fbeecheii]]
(* for M. beecheii using avg value for b *)
```

0.113234

```
ESS[0.75, 0.5, bavg, ffavosa, μfavosa, wfromψ[ψfavosa, ffavosa]]
(* for M. favosa using avg value for b *)
```

0.0758988

```
ESS[0.75, 0.5, bavg, favg, μavg, wfromψ[ψavg, favg]]
```

0.0996809

SENSITIVITY ANALYSIS

As expected, higher values of per capita rate of brood cell construction b will translate in a smaller colony-level cost of queen overproduction, and a higher ESS level of queen production :

```
Plot[{100 * ESS[0.75, 0.5, b, favg, μavg, wfromψ[ψavg, favg]]},
 {b, 0.03, 0.1}, AxesOrigin → {0.03, 0}, Frame → True,
 FrameLabel → {"Nr of new cells produced per day per worker (b)" ,
   "ESS queen production (%)"},
 PlotStyle -> {Black}]
```

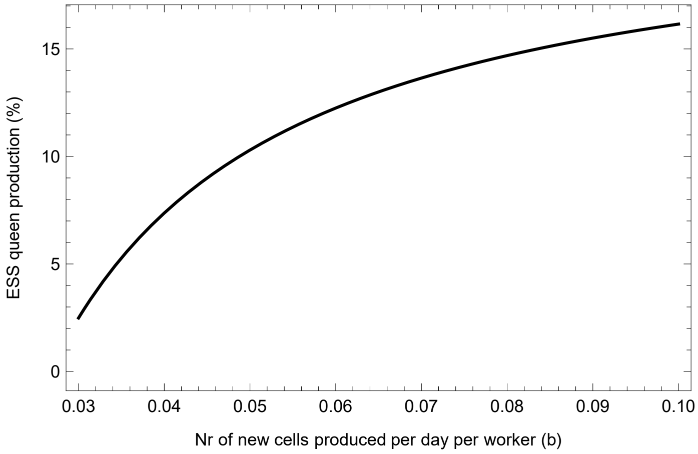

As expected, if a higher fraction of the queen's eggs are female, the colony-level cost of queen overproduction will be reduced, as the colony will then produce relatively more workers' to sustain itself, and the ESS level of queen production will be higher. In addition, when most of the males are workers' sons, the colony-level cost of queen overproduction overproduction is higher and the inclusive fitness cost of queen overproduction is higher (due to the greater colony-level productivity cost as well as the greater relatedness to the males that are reared) and the ESS level of queen production lower (blue line) than when all the males are sons of the queen (red line). This effect is partly compensated, however, by the fact that the proportion of eggs that the queen fertilises *f* and levels of worker reproduction show two alternative equilibria, with workers either producing most of the males and the queen fertilising most of the eggs that she lays or the workers not reproducing and the queen fertilising a much smaller proportion of her eggs (Wenseleers et al. 2013) :

```
Plot[
 {100 * Clip[ESS[0.75, 0.5, bavg, f / 100, μavg, wfromψ[ψavg, f / 100]], {0, 1}],
  100 * Clip[ESS[0.75, 0.5, bavg, f / 100, μbeecheii,
     wfromψ[ψbeecheii, f / 100]], {0, 1}],    100 *
   Clip[ESS[0.75, 0.5, bavg, f / 100, μfavosa, wfromψ[ψfavosa, f / 100]], {0, 1}]},
 {f, 75, 100}, AxesOrigin → {75, 0}, Frame → True, FrameLabel →
  {"Queen-laid eggs that are female (f) (%)", "ESS queen production (%)"},
 PlotStyle -> {Black, Red, Blue}]
```

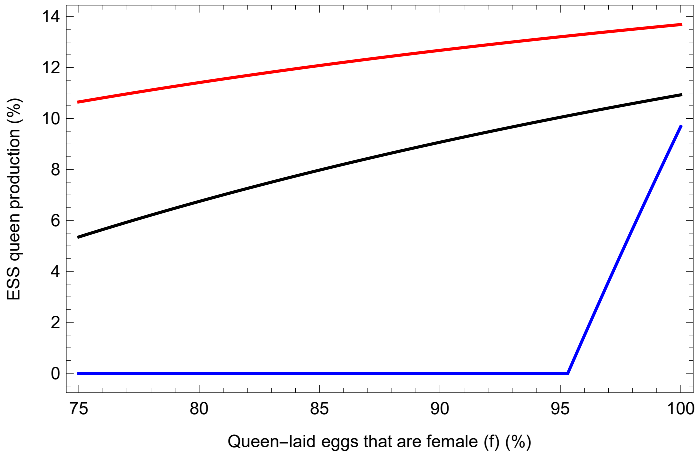

As expected, if workers have a longer life expectancy ($1/\mu$) and lower mortality rate μ, the colony-level cost of queen overproduction will be reduced, and the ESS level of queen production will be higher :

```
Plot[{100 * ESS[0.75, 0.5, bavg, favg, 1 / l, wfromψ[ψavg, favg]]},
 {l, 30, 50}, AxesOrigin → {30, 0}, Frame → True,
 FrameLabel → {"Worker life expectancy (days)", "ESS queen production (%)"},
 PlotStyle -> {Black}]
```

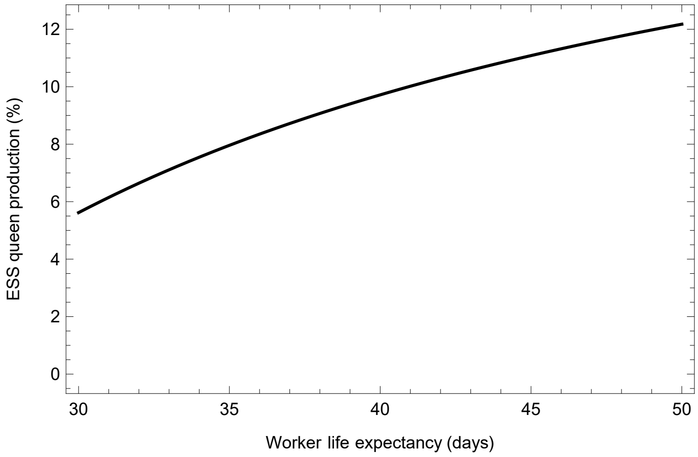

Supplement: qrad068_suppl_Supplementary_Material [file qrad068_suppl_supplementary_material.zip › caste fate conflict model_FINAL.pdf]
